# Transcripts of repetitive DNA elements signal to block phagocytosis of hematopoietic stem cells

**DOI:** 10.1126/science.adn1629

**Published:** 2024-09-13

**Authors:** Cecilia Pessoa Rodrigues, Joseph M. Collins, Song Yang, Catherine Martinez, Ji Wook Kim, Chhiring Lama, Anna S. Nam, Clemens Alt, Charles Lin, Leonard I. Zon

**Affiliations:** 1. Howard Hughes Medical Institute, Boston Children’s Hospital Boston, MA, USA.; 2. Harvard Stem Cell Institute, Stem Cell and Regenerative Biology Department, Harvard University, Cambridge, MA, USA.; 3. Department of Pathology and Laboratory Medicine, Weill Cornell Medicine, New York, NY, USA.; 4. Wellman Center for Photomedicine, Mass General Research Institute, Boston, MA, USA; 5. Center for Systems Biology, Massachusetts General Hospital, MA, USA.

## Abstract

Macrophages maintain hematopoietic stem cell (HSC) quality by assessing cell surface Calreticulin (Calr), an “eat-me” signal induced by reactive oxygen species (ROS). Using zebrafish genetics, we identified Beta-2-microglobulin (B2m) as a crucial “don’t eat-me” signal on blood stem cells. A chemical screen revealed inducers of surface Calr that promoted HSC proliferation without triggering ROS or macrophage clearance. Whole genome CRISPR-Cas9 screening showed that Tlr3 signaling regulated *b2m* expression. Targeting *b2m* or *Tlr3* reduced the HSC clonality. Elevated B2m levels correlated with high expression of repetitive elements (RE) transcripts. Overall, our data suggest that RE-associated dsRNA could interact with TLR3 to stimulate surface expression of B2m on HSPCs. These findings suggest that the balance of Calr and B2m regulates macrophage-HSC interactions and defines hematopoietic clonality.

## Graphical abstract

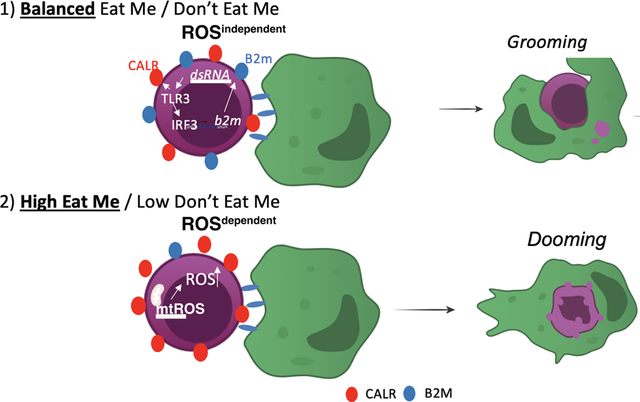

## Introduction

Development, tissue integrity, and defense against immunogenic challenges necessitates the effective removal of damaged and impaired cells (1, 2). Innate immune cells such as macrophages and neutrophils orchestrate the removal or phagocytosis of these dying cells by discriminating between molecules expressed on their surface (2). Therefore, the phagocytic process needs to be carefully regulated to avoid the unwarranted elimination of healthy cells (2). Surface molecules, such as complement opsonins, exposed phosphatidylserine (PS), Calreticulin (Calr) and annexin I serve as signals to initiate the phagocytosis (“eat-me” signal)(2). These surface molecules are not present at high levels on the surface of living healthy cells, except during specific physiological events. On the other hand, surface molecules such as B2m serve as “don’t eat-me” signals to prevent the unwarranted clearance of healthy cells that concurrently present “eat-me” molecules on their surfaces (3, 4).

Hematopoietic stem cells that originate during embryonic development sustain lifelong tissue homeostasis (5) and phagocytosis by macrophages plays a pivotal role in ensuring the quality of newly formed hematopoietic stem and progenitor cells (HSPCs) within the embryonic niche (6). Hematopoietic stem and progenitor cells (HSPCs) present surface Calreticulin (Calr) as an “eat-me” signal that induces macrophage interaction. During their interaction, a macrophage may completely engulf a HSPC (dooming), or sample a small portion of the HSPC cellular material without killing it (grooming) (6). While HSPCs dooming eliminates a selected stem cell clone, the HSPCs grooming likely regulates HSPCs proliferation by activating Il-1b-dependent signaling (6). Grooming-and-dooming is an important quality control step that removes stressed HSPCs during development. Despite the importance of these processes, the mechanism by which macrophages distinguish which HSPCs to doom and which HSPCs to groom remains elusive. Through live imaging and *in vivo* cellular barcoding techniques, we demonstrated that the balance between “eat-me” and “don’t eat-me” signals is provided by reactive oxygen species (ROS) and toll-like receptor 3 (Tlr3) activation, respectively. These signals determine the number of long-lived HSC clones that contribute to the adult blood system through macrophage-mediated quality assurance mechanisms.

## Results

### Calr expression is induced by processes associated with and without reactive oxygen species

**Fig.1.**
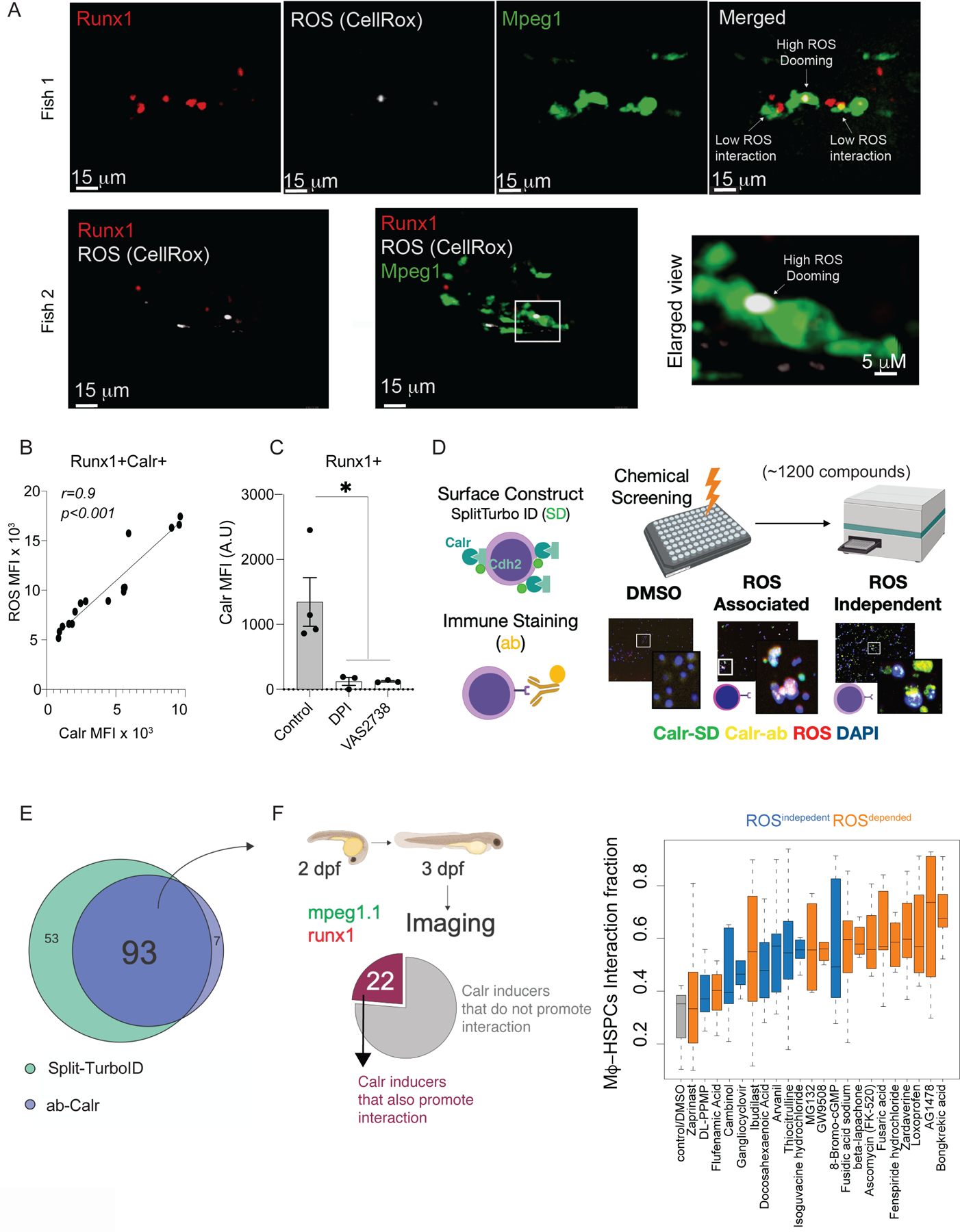
Chemical screen identifies ROS^dependent^ and ROS^Independent^ surface Calr-inducers. (A) Representative imaging from 3 independent experiments showing that ROS+Runx1+ cells are preferentially doomed by the macrophages. Green: Mpeg; Red: Runx1; white: CellRox probe. (B) ROS levels in embryonic HSPCs marked by surface Calreticulin (Calr). Data were analyzed by Pearson correlation. MFI, median fluorescence intensity. A.U, arbitrary unit. Data points represent a pool of 100–300 3 dpf embryos (C) Surface Calr levels on HSPCs following ROS inhibition with diphenylene (DPI) or VAS2870 (Nox inhibitor). Data were analyzed by Kruskall-Wallis test followed by Dunn’s; *P <0.05. DMSO, dimethyl sulfoxide Data points represent a pool of 100–300 3 dpf embryos. Data are means ± SEM (A-C) were performed in a pool of 100–300 zebrafish embryos in 2 independent experiments. (D) A schematic overview of the chemical screen: Two approaches were employed to ensure that our system specifically recorded the surface Calreticulin values 1) HEK293 cells were transfected with the SPLIT-TURBO ID where the C-Terminus was design to carry Cdh2, a membrane protein, while the N-terminus was carrying Calr, and 2) Anti-Calr was conjugated with a A647 using the Zenon technology. Next, the cells were plated in 384-well plates and treated with a panel of 1200-bioactive small molecules in 3 different concentrations (5uM, 2.5uM, and 0.625 uM). The cells were also labeled with a ubiquitous ROS probe (CellRox) and DAPI for nuclear staining. After 24 hrs the Zeiss Cell Discoverer 7 (CR7) microscope was used to read the plates. Data were analyzed using the CellProfiler and R-Sight HTS software. SD, Split-turbo ID. ab, Calreticulin antibody. DMSO, dimethyl sulfoxide. (E) Numbers and overlap in compounds that induced surface Calreticulin in HEK293 for both systems. Each compound was tested in replicates and 2–4 random fields of view were chosen for acquisition. The same well was used for the SPLIT-Turbo, Ab-Calr and CellROX acquisition. (G) Left panel: Number of compounds that increased surface Calreticulin in vitro and in vivo (zebrafish embryos). Right panel: Live image microscopy was used to determine the Interaction fraction between macrophage-HSPCs using *mpeg-GFP;runx1-mCherry* embryos that label macrophage and HSPCs, respectively. Highlighted in blue are the ROS^indepedent^ and black represent ROS^dependent^ compounds. Data are means ± SEM. Calr, surface Calreticulin. ROS, Reactive oxygen species. The zebrafish live imaging experiments performed to determine the macrophage-HSPCs interaction were conducted as two independent experiments and a minimum of 5 embryos were imaged.

Stress associated with reactive oxygen species (ROS) within HSPCs mediates the surface presentation of “eat-me” molecules as Calreticulin (Calr)(6). This stress in HSPCs correlates with FoxO signaling, known to mediate cellular detoxification from spurious ROS (7). Similar to the proposed quality control mechanism, we observed that HSPCs with high ROS were removed (doomed) by macrophages, while low ROS HSPCs were not doomed, but rather prompted to continue dividing after interacting with macrophages (Fig. 1A, movie S1). We evaluated ROS levels in zebrafish HSPCs and confirmed that increased levels of ROS correlated with surface presentation of the “eat-me” signal, Calr (Fig. 1B, Fig S1A). Conversely, when we reduced ROS in HSPCs by treating zebrafish embryos with the ROS scavengers diphenyleneiodonium (DPI) or VAS2738 (VAS) (8), we observed low levels of surface Calr (Fig. 1C) and reduced macrophage interactions (6) with stem cells. This showed that ROS levels were indicative of higher surface Calr and presumably macrophage-HSPCs interaction.

Nonetheless, the intrinsic mechanism regulating surface Calr in a ROS^independent^ manner remains unknown. To evaluate the pathways in HSPCs that trigger surface Calr presentation systematically, we targeted a myriad of signaling pathways in HEK293 cells by screening a panel of 1,200 bioactive small molecules (Fig. 1D). Calr is an abundant chaperone in the endoplasmic reticulum. Thus, to evaluate only Calr on the cell surface, we designed a SPLIT-TURBO ID (9) construct targeting the association of Calr and a membrane protein, Cadherin 2 (CDH2). We orthogonally evaluated Calr surface presentation with a fluorescent Calr antibody (Zenon-Technology). Through these two independent approaches, we identified compounds that robustly increase surface Calr presentation Concurrently, we also labeled cells with a ubiquitous ROS probe (CellRox) to identify compounds that induce surface Calr in a ROS-associated manner (Fig. 1D and fig. S1B and S1C). We found 93 compounds that increased surface Calr with a robust dose-dependent response in cells (Fig. 1E), hereafter referred to as “Calr-inducers”. Among the 93 Calr-inducers, 54 were associated with increase of ROS (ROS^dependent^), while 39 did not affect ROS levels (ROS^independent^) (Data File S1). DMSO was used as the vehicle control, while DPI and H_2_O_2_ were used as internal negative and positive controls, respectively. Using the Chemical Annotation Toolkit (10), we analyzed the known biological function of ROS^dependent^ compounds. As expected, we found a strong positive correlation with cellular stress pathways, such as oxidation by cytochrome P450 and DNA damage, while ROS^independent^ chemicals were not enriched in a given pathway (Data File S1).

To test the effect of the 93 Calr-inducers on macrophage-HSPC interaction outcomes, we used *runx1+23:mCherry; mpeg1.1:EGFP* zebrafish embryos, expressing fluorescent reporters in HSPCs and macrophages, respectively (11). At 48 hours postfertilization (hpf), embryos were exposed to the 93 Calr-inducers (Fig. 1F). After 24 hrs of exposure, we evaluated macrophage-HSPC interactions by live cell imaging. Twenty-two of 93 Calr inducers facilitated macrophage-HSPC interactions above baseline levels (Fig. 1F and Data File S1). We confirmed increased surface Calr presentation by these compounds (fig. S1D).

Both ROS^dependent^ and ROS^independent^ compounds increased macrophage-HSPC interaction ratios by a similar level (fig. S1E). We evaluated apoptosis by Annexin-V staining on cells from wildtype 72 hpf embryos. The 22 compounds did not change apoptosis levels compared to DMSO treated embryos, except for the ROS^dependent^ β-Lapachone (Fig. 1G and fig. S1F). Thus, Calr-inducers triggered surface Calr independently of an apoptotic/cell death pathway (fig. S1D-F). Next, to validate the dependence of Calr in mediating the increased macrophage-HSPCs interaction observed upon the 22-Calr-inducers treatment (Fig. 1G), we generated mosaic deletions (crispants) of a *Calr* isoform in zebrafish that regulates macrophage-HSPC interactions, *Calr3b* (6). We found that the 22 Calr-inducers facilitated the interaction in a *Calr3b*-dependent manner (fig. S1G-H). Collectively, these results demonstrate that surface Calr level determines macrophage-HSPC interaction. Both ROS-dependent and ROS-independent pathways increase surface Calr presentation, suggesting that various sources of stress lead to the presentation of “eat-me” signals.

### Macrophage “dooming” or “grooming” behavior is determined by HSPC ROS levels

**Fig.2:**
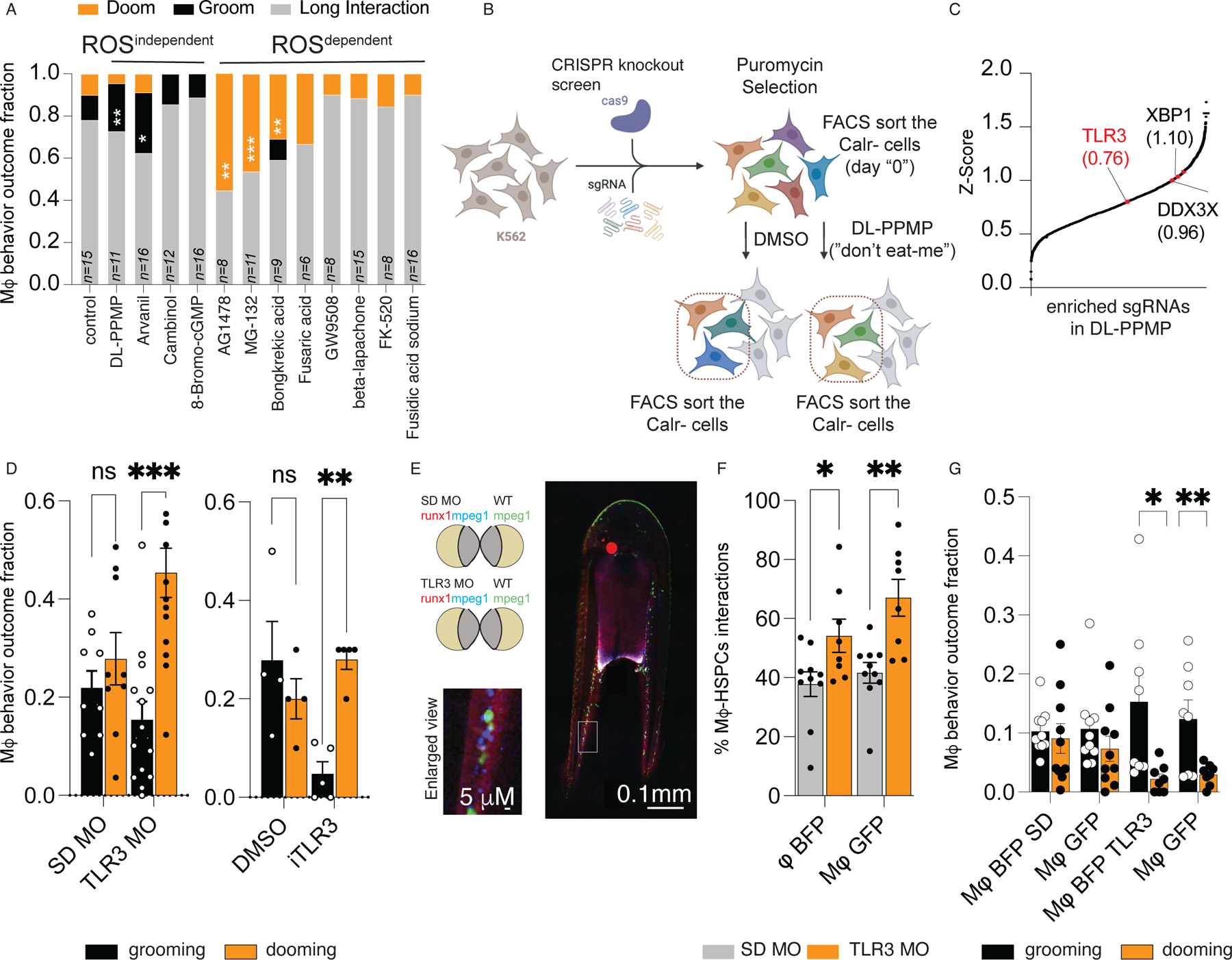
Tlr3 is required for HSPC grooming. (A)Stack Plot showing the macrophage interaction behavior in the presence of the Calr-inducers. rGrooming behavior is shown in the black bar, while dooming behavior as the orange bar. The imaging was conducted using 3 dpf zebrafish embryos. The exact sample size is indicated in the figure, *n*=6–16 from 2 independent experiments. Data were analyzed by Wilcoxon matched-pairs signed rank test. *P=0.0156, **P=0.002, ***P=0.001. (B) A scheme overview of the Gecko 2.0 CRISPR/Cas9 knockout screen. (C) sgRNAs significantly enriched (*P*<0.05) were identified using the MAGECK software and plotted as cumulative stack based on their Z-Score. The arrows indicate examples of significant enriched sgRNAs and their Z-Score depicted inside the brackets. The screen was conducted using K562 cells. The screen was performed in 3 independent experiments. (D) Knock-down of *Tlr3* (Tlr3 MO) or iTlr3 treatment reduces the fraction of HSPCs that are groomed, but increases the fraction of HSPCs that are doomed in 3 dpf zebrafish embryos. The macrophage behavior percentage outcome was calculated by counting the total number of interacting macrophages and dividing by the total number of dooming or grooming events. Data points represent an image embryo, SD MO *n*=9, TL3 MO *n*= 11, DMSO *n*=4 and iTLR3 *n*=5. Experiments were performed in 2 independent experiments. Two-way ANOVA followed by Sidak’s multiple comparison. **P=0.0027; ***P=0.00005. (E) Representative imaging showing the experimental design of the parabiosis experiment. (F) Both macrophages showed higher interaction rates with the HSPCs. *P=0.04, **P=0.0015. Data were analyzed using a two-way ANOVA test. The macrophage-HSPCs interaction percentage was calculated by counting the total number of HSCPs (runx1+ cells) and dividing by the total number HSCPs interacting with a macrophage. (G) Bar-plot showing the macrophage behavior outcome fraction. The macrophage behavior percentage outcome was calculated by counting the total number of interacting macrophages and dividing by the total number of dooming or grooming events. *P= 0.01, **P=0.0057. Data were analyzed using an unpaired Student t-test. (F-G). Data points represent an image embryo, *n*= 10 from 3 independent experiments.

Since antioxidant treatment hinders the surface Calr levels of human HSPCs exposed to ROS^dependent^ compounds (Fig S1I), we reasoned that compounds that promoted dooming would be associated with higher ROS levels due to mitochondrial dysfunction. Supporting our hypothesis, ROS^dependent^ compounds that promoted only dooming (Fig. 2A) also impaired mitochondrial membrane potential (fig. S1J-S1L) and absence of macrophage-HSPCs interactions induced higher accumulation of mitochondrial ROS (fig. S1M-N), thus supporting quality control ensured by macrophages.

In contrast, ROS^independent^ compounds increased HSPC proliferation, which was in line with increased grooming behavior (Fig. 2A, fig. S2A). Taken together, these results suggested that surface Calr presentation on HSPCs induce macrophage interaction, but the outcome of that interaction was determined by HSPC cellular ROS levels. ROS^dependent^ compounds stimulated dooming behavior and HSPC death. ROS^independent^ compounds stimulated grooming behavior and HSPC proliferation.

The pro-proliferative effect of Calr-inducing compounds on HSPCs could be an indirect consequence of impaired intrinsic macrophage behavior. Therefore, we tested the chemotaxis and phagocytic activity of macrophages treated with ROS^independent^ compounds in vitro. To evaluate phagocytosis, we treated RAW-274 macrophages with the Calr-inducers. After exposure for 24 hrs, we added GFP-rhodo zymosan, a pathogen particle that promotes phagocytosis, to the culture and measured the number of zymosan-GFP+ macrophages by live cell imaging. We did not observe impaired zymosan phagocytosis in the treated cells. LPS was used as a positive macrophage stimulator (fig. S2B). We evaluated macrophage chemotaxis with an agarose-chemotaxis assay (12). RAW-274 cells were treated with DMSO (negative control), LPS (positive control) or the ROS^independent^ compound. The chemoattractant spot was filled with a medium containing LPS as bait for macrophage chemotaxis, which was not affected by this treatment (fig. S2C). These results suggested ROS^independent^ Calr-inducers did not alter intrinsic macrophage function. Thus, ROS^independent^ compounds drove macrophage-mediated HSPC grooming without inducing macrophage autonomous effects (Fig. 2A).

### Toll-like receptor 3 induces surface Calr in a pro-grooming context

In order to determine how ROS^independent^ compounds induced surface Calr presentation to promote macrophage grooming, rather than dooming, we conducted a genome-wide CRISPR screen. We chose the ROS^independent^ compound DL-PPMP as the Calr-inducer, as it led to high surface Calr (Fig S1C), macrophage-HSPC interactions (Fig. 1F), HSPC proliferation (fig. S2A), and increased the macrophage grooming ratio (Fig. 2A, movie S2). To define the intricate network required for surface Calr expression, we used lentiviral delivery of a knockout library that targeted 18,080 genes with 64,751 unique guide sequences (13). We treated K562 human leukemia cells with DL-PPMP or DMSO (control) and flow cytometry sorted for Calr-negative cells (Fig. 2B). Here, by sorting for Calr-negative cells and enriching for the sgRNA in those cells, we determined which genes regulate surface Calr presentation in response to the Calr-inducer in the “don’t eat-me” context.

We focused on the genes that were specifically enriched in the DL-PPMP stimulated cells (Fig. 2C and Data File S2). Amongst the targets, we found enrichment of genes that upregulate Calr presentation, associated with cytosolic DNA/RNA sensing and viral immune responses. Namely, Tlr3, a dsRNA sensor, DDX3X, a RNA helicase, and XBP1, a protein that among other terms regulates MHC class II genes and is a key transcription factor regulator of ER stress (14–16). In validation experiments, Tlr3 depletion reduced surface Calr in K562 cells (fig. S2G), indicating that cytosolic DNA/RNA may mediate the increase in surface Calr.

In parallel, we performed a whole genome CRISPR-Cas9 screen of K562 cells treated with a ROS^dependent^ compound that promoted HSPC dooming, Bongkrekic acid. While we scored enrichment of Calr, confirming the targeting efficiency of our screen, we did not find enrichment of Tlr3 in this dataset (Data File S2). This data supported the hypothesis that a pro-grooming surface Calr presentation is mediated by Tlr3.

### Tlr3 signaling in HSPCs is required for macrophage-mediated grooming.

We sought to evaluate the biological relevance of Tlr3 in HSPCs for controlling dooming versus grooming by macrophages. We tested this with two orthogonal approaches. We depleted *Tlr3* by injecting a morpholino in single-cell zebrafish embryos (termed “morphants”, fig. S2H) or we pharmacologically inhibited Tlr3 by treating zebrafish embryos with the dsRNA/Tlr3 inhibitor (fig. S2I), CUCPT4a (iTlr3) (17). We treated or depleted after 48 hours since the emerging/budding of HSPCs from the ventral wall of the dorsal aorta (VDA) reliably occurs during the first 48 hpf (18). In both approaches, we observed a reduced number of HSPCs presenting surface Calr *in vivo* by flow cytometry (fig. S2H-I). These data confirmed that Tlr3 is required for surface Calr presentation on the surface of HSPCs *in vivo*.

We then tested if morpholino-depletion of Tlr3 or iTlr3 inhibition modulated macrophage-HSPC interactions and interaction outcomes. Despite a reduction in Calr+HSPCs, *Tlr3* depletion by morpholino or iTlr3 increased dooming and decreased grooming (Fig. 2D, movie S3-S5). To address potential cell autonomous defects in macrophage behavior, we performed parabiosis experiments where standard or *Tlr3* morpholino-injected embryos from the *runx1+23mCherry*;*mpeg1*:BFP were fused to a *mpeg.1:EGFP* wild type zebrafish (Fig, 2E). Macrophages of both origins, wild type (green) and *Tlr3-*morphants (blue), equally showed increased engagement to HSPCs followed by increased dooming behavior. This demonstrated that Tlr3 acts in a HSPC-autonomous manner to prevent dooming behavior (Fig. 2F, 2G). To investigate if Tlr3 was sufficient to promote grooming, we sorted murine Lineage^-^Sca1^+^cKit^+^ (HSPCs, LSK^+^) cells, pretreated them with either Poly I:C, a dsRNA mimic (Tlr3 agonist) (19), or DMSO, and cocultured them with their autologous bone marrow-derived macrophages (BMDM). We observed that poly I:C-pretreated HSPCs cells showed higher grooming ratios (fig. S2J-K), ergo suggesting that Tlr3/dsRNA signaling in HSPC promoted grooming behavior.

### Tlr3 signaling directs HSPC surface presentation of B2m

To gain insight into the molecular mechanism triggered by the “don’t eat-me” compound, DL-PPMP, we analyzed previously available bulk RNA-seq data from a DL-PPMP-treated human cancer cell line (20). We identified 2,285 upregulated genes in treated cells, enriched for pathways associated with the viral immune response, such as regulation of interferon-alpha production, antigen processing, and presentation of endogenous peptides via MHC class I. Consistent with our data, K562 cells treated with DL-PMPP expressed higher levels of Beta 2 microglobulin (B2M), a molecule required for the MHC-I stabilization on the cell surface (fig. S3A). Although MHC-I is required for antigen presentation and CD8^+^ T cell activation (21), it can also prompt a “don’t eat-me” signal (22). For example, MHC class I-B2M expression protects cancer cells from phagocytosis via engagement of LILRB1/LILRB2 expressed in immunosuppression-related cells, such as tolerogenic dendritic cells (DCs) and M2-type macrophages (22–24).

Evolutionary, this mechanism might be conserved because based on data from Actinopterygii, the LILR system originated 450 million years ago, in which the leukocyte immune-type receptor (LITR) shows orthologous relationship to human LILR receptor (25, 26). Based on our CRISPR-screen to identify the molecular cues of surface Calr inducers in the “don’t eat-me” context (Fig. 2C) and results showing that Tlr3 facilitated grooming (Fig.S2J-K), we hypothesized that HSPCs would display more B2m in response to Tlr3 signaling, thereby suppressing macrophage phagocytosis and preventing dooming behavior. To test this hypothesis, we evaluated surface levels of B2m on HSPCs in Tlr3 zebrafish morphants. We found that depleting *tlr3* in zebrafish embryos resulted in fewer B2m^+^ HSPCs (fig. S3B). Considering that Tlr3 zebrafish morphants showed higher dooming events (Fig 2D) our results suggested that the lower B2m expression, resulting from lower Tlr3 expression, biased the HSPCs to be doomed by the macrophages.

### Surface B2m determines the outcome of macrophage-HSPC interactions mediated by Calr

We treated 48 hpf zebrafish embryos with the ROS^independent^ compounds and evaluated the surface B2m on HSPCs by FACS. We found that ROS^independent^ compounds increased B2m levels (fig. S3C,D; blue), while “eat-me” or iTlr3 treatments decreased surface B2m levels (fig. S3C,D). To validate whether the signal input provided by Calr and B2m was important for the outcome of macrophage-HSPC interactions, we used CRISPR/Cas9 to generate zebrafish crispant embryos with mosaic deletions of *b2m* and treated them with ROS^independent^ Calr inducers. DMSO was used as the vehicle control in the embryos injected with control sgRNA and *b2m* sgRNA. Upon *b2m* depletion, ROS^independent^ Calr inducers promoted dooming, rather than grooming (fig. S3E). Together, these data supported the hypothesis that ROS^independent^ Calr-inducers promoted grooming via both Calr and B2m surface presentation, in which Calr promotes the macrophage-HSPC interaction and B2m provides the “don’t eat-me” signal hindering HSPCs from being fully engulfed by macrophages.

**Fig.3:**
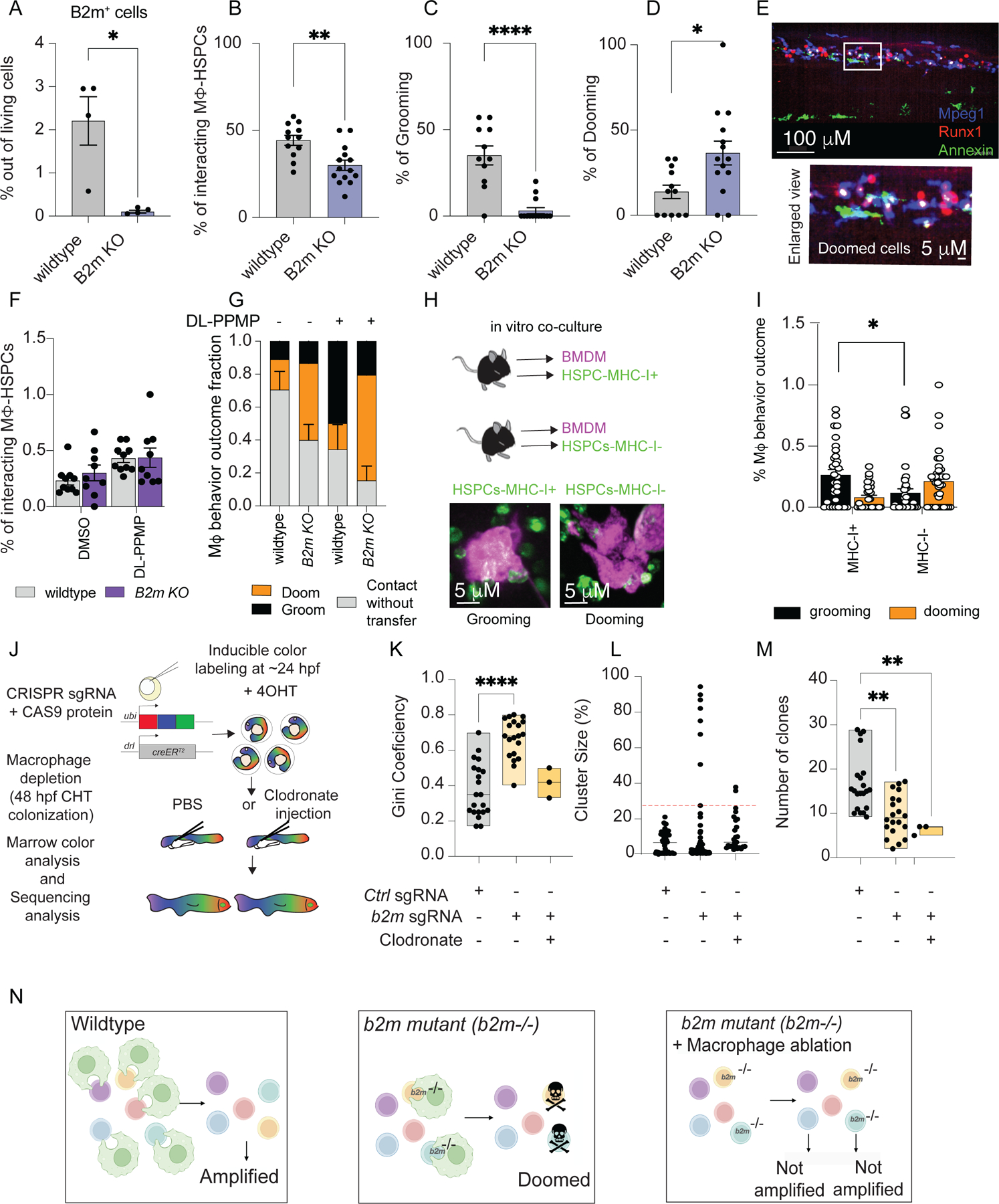
B2m acts as a “don’t eat-me” molecule and prevails unwarranted HSPCs dooming. (A) *b2m* knock-out stable mutants (*B2m KO*) showed significant reduction in B2M levels. Data were analyzed by unpaired Mann Whitney test. *P=0.028. Data points representing a pool of 100 embryos (B)Barplot showing the interaction percentage. *b2m* KO embryos showed significant lower macrophage-HSPCs interaction with a (C-D) dooming dominance. Data were analyzed by unpaired Mann-Whitney. *P=0.012, **P=0.0021, ****P<0.0001. Data points represent an image embryo. Wildtype n=12 and B2m KO n=14. This data represents 3 independent experiments. The macrophage-HSPCs interaction percentage was calculated by counting the total number of HSCPs (runx1+ cells) and dividing by the total number HSCPs interacting with a macrophage. (E) Representative imaging showing the increased dooming behavior observed in the *B2m* homozygous mutant. *n*=4 from 2 independent experiments (F) Bar-plot showing the interaction rates in wildtype and *B2m* KO zebrafish embryos, *n*=4. Data points depict the field of view. (G) The macrophage behavior percentage outcome was calculated by counting the total number of interacting macrophages and dividing by the total number of dooming or grooming events. We found that while in the wildtype background DL-PPMP promotes grooming, in the *B2m* KO background it promotes dooming. (H) A scheme overview of the murine HSPCs (LSK+) and macrophage co-culture. Left lower panel is a representative imaging showing the sorted murine HSPC-MHC-I^+^ (Green) being groomed by a macrophage (magenta), while on the right lower panel is a murine HSPC-MHC-I^-^ being doomed by a macrophage. (I) MHC-I^+^ HSPCs showed higher grooming rates compared to MHC-I^-^ cells. Data were analyzed using non parametric one-way ANOVA followed by Kruskall Wallis. *P=0.03. Data are means ± SEM. The macrophage behavior percentage outcome was calculated by counting the total number of interacting macrophages and dividing by the total number of dooming or grooming events. (J) A schematic overview of the *Zebrabow-M* system: Animals with 15 to 20 insertions of a multicolor fluorescent cassette are crossed to the *draculin:CreERT2* line to enable clonal labeling of lateral plate mesoderm lineages. By treating with 4-hydroxytamoxifen (4-OHT) at 24 hpf just after HSC specification, individual stem cell lineages express specific fluorescent hues that can be quantified in the adult marrow. Families of *Zebrabow-M;draculin:CreERT2* animals injected with *b2m* morpholino with or without clodronate liposomes exhibit (K) reduced numbers of HSC clones. (L-M) Clonal dominance in the adult marrow. Clodronate liposome was injected at 48 hpf. Data points represent an adult kidney marrow. Control n= 22, b2m sgRNA n=21 and clodronate n=3. Experiments were conducted at least 2 times. (A, B, C, D,F,G,I,K,L,M) Data are means ± SEM. Experiments were conducted in vivo using zebrafish as a model. (N) A schematic overview of the clonal diversity in regards to the “don’t eat-me” signal.

Mosaic crispants are limited by editing efficiency. Thus, to confirm the role of B2m in macrophage-mediated HSPC grooming, we generated *b2m* stable knockout zebrafish. We used CRISPR/Cas9 to cause a frameshift mutation in the 3-exon of the *b2m* gene in zebrafish with a *runx1+23:mCherry;mpeg1-EGFP* background. We investigated the outcome of macrophage-HSPC interactions in these homozygous mutants by live cell imaging at 72 hpf. *B2m* depletion promoted HSPC-dooming and decreased HSPC-grooming (Fig. 3A–3E and fig. S3D), which further supported the role of surface B2m as a “don’t eat-me” signal on HSPCs. This increase in dooming was not associated with increased numbers of Calr^+^ HSPCs (fig. S3G). Also, despite the low surface Calr on HSPCs surface we observed fewer Runx1^+^ cells in the caudal hematopoietic tissue (CHT)(fig. S3H), potentially indicating enhanced HSPCs dooming.

Next, to validate the role of B2m as a “don’t eat-me” signal, we treated our *b2m* knockout embryos with the ROS^independent^ compound, DL-PPMP. We rationalized that DL-PPMP would increase surface Calr (increasing macrophage-HSPC interactions), but in the absence of B2m, the interactions would lead to dooming. We observed that DL-PPMP stimulated interactions in both wild type and *b2m* knockout embryos. However, b2m knockout HSPCs were doomed, while wild-type HSPCs were groomed (Fig. 3F–3G). These results supported the importance of surface B2m to instruct grooming behavior by the macrophages.

We then sought to evaluate whether the “don’t eat-me” signal mediated by B2m was conserved throughout evolution, thus we sorted murine HSPCs (LSK^+^) that were MHC-I^+^ or MHC-I^-^ and co-culture them with bone marrow derived macrophage (BMDM) for 4 hours (Fig. 3H). We opted to sort for class I MHC because its surface expression is dependent on the presence of B2M. We found that murine MHC-I^+^ HSPCs promoted macrophage grooming (Fig. 3I). Overall, these results showed that B2m decorates the surface of HSPCs and promotes the “don’t eat-me” signal during the macrophage-HSPCs interactions.

### B2m is required for safeguarding HSC clonal complexity

As in B2m germline mutations in mammals (27), we did not observe major changes in development following B2m knockout in adult zebrafish, except for a decrease in lymphoid cells (fig. S3I), possibly reflecting impaired CD8^+^ differentiation (28).

HSC clonal complexity is essential for maintaining a functional and resilient immune system, supporting long-term hematopoietic function, and reducing the risk of hematological diseases (29, 30). Since grooming and dooming regulates HSC clonal complexity (6), we sought to determine if B2m depletion could impact the HSC clonal landscape. We hypothesized that increased dooming in *b2m* mutants reduces the number of HSC clones in adulthood. To test this, we generated mosaic deletions using tissue editing with inducible stem cell tagging via recombination (TWISTR) to combine CRISPR/Cas9-mediated gene editing with Zebrabow (zbow) HSC color labeling (31, 32). This allows mutant and wild type stem cells to compete *in vivo*. *Zebrabow-M;draculin:CreERT2* embryos permit unique lineage labeling of individual HSC clones at 24 hpf (Fig. 3J). Mosaic deletion of *b2m* reduced the number of myeloid and lymphoid/progenitor clones (fig. S3J) and promoted clonal dominance (Fig. 3K–3M, fig. S3K). Additionally, macrophage ablation before HSPC lodgment in CHT rescued clonal dominance in *b2m* crispants (Fig. 3K–3M), but did not rescue clone numbers (Fig. 3M). The decrease in clone numbers may reflect the impairment of interleukin 1β (Il-1β)-driven proliferation (6), therefore limiting the number of HSC clones (Fig. 3N). Considering the mosaic nature of TWISTR, we hypothesized that the more abundant (or dominant) clones found in the *b2m* mutation condition are wild type cells that do not carry *b2m* mutation. We, therefore, sorted dominant and non-dominant clones and evaluated their *b2m* editing efficiency. Indeed, dominant clones were wild type for *b2m*, suggesting that *b2m* mutant clones were doomed and led dooming-resistant wildtype clones to overtake the adult marrow (fig. S3K). These results indicate that B2m decorates the surface of HSPCs and protects them against macrophage removal thus affecting the HSPCs clonality in adulthood.

Irf3 mediates a Tlr3/Tlr4-specific antivirus gene program upstream of *b2m* expression (33–35). We therefore used the TWISTR system to generate TWISTR-*Irf3* mutants to elucidate the molecular pathways that regulate HSC clonality via B2m. We also generated TWISTR-*Tlr3* mutants and treated zebrafish embryos with iTlr3 to suppress Tlr3/dsRNA downstream signaling at 48 hpf. Like *b2m*-mutants, mosaic depletion of *Irf3* and *Tlr3*, as well as inhibition of Tlr3 signaling, collectively reduced the number of myeloid clones while increasing clonal dominance (fig. S3L–Q). The TWISTR-*Irf3* mutants similarly showed wild-type clonal dominance, indicating a competitive disadvantage of *Irf3*-depleted stem cells (fig. S3L-S3N). Collectively with the imaging results, this shows that both B2m mutant HSCs, and also Irf3 mutant HSCs, are removed by macrophages because the cells no longer present the “don’t eat-me” signal. These data suggest an operative molecular network of Tlr3 activation cascades that promote Irf3 activation (36), which could promote the transcription of *b2m*.

### Cytosolic dsRNAs promote B2m expression and protection from dooming

**Fig.4.**
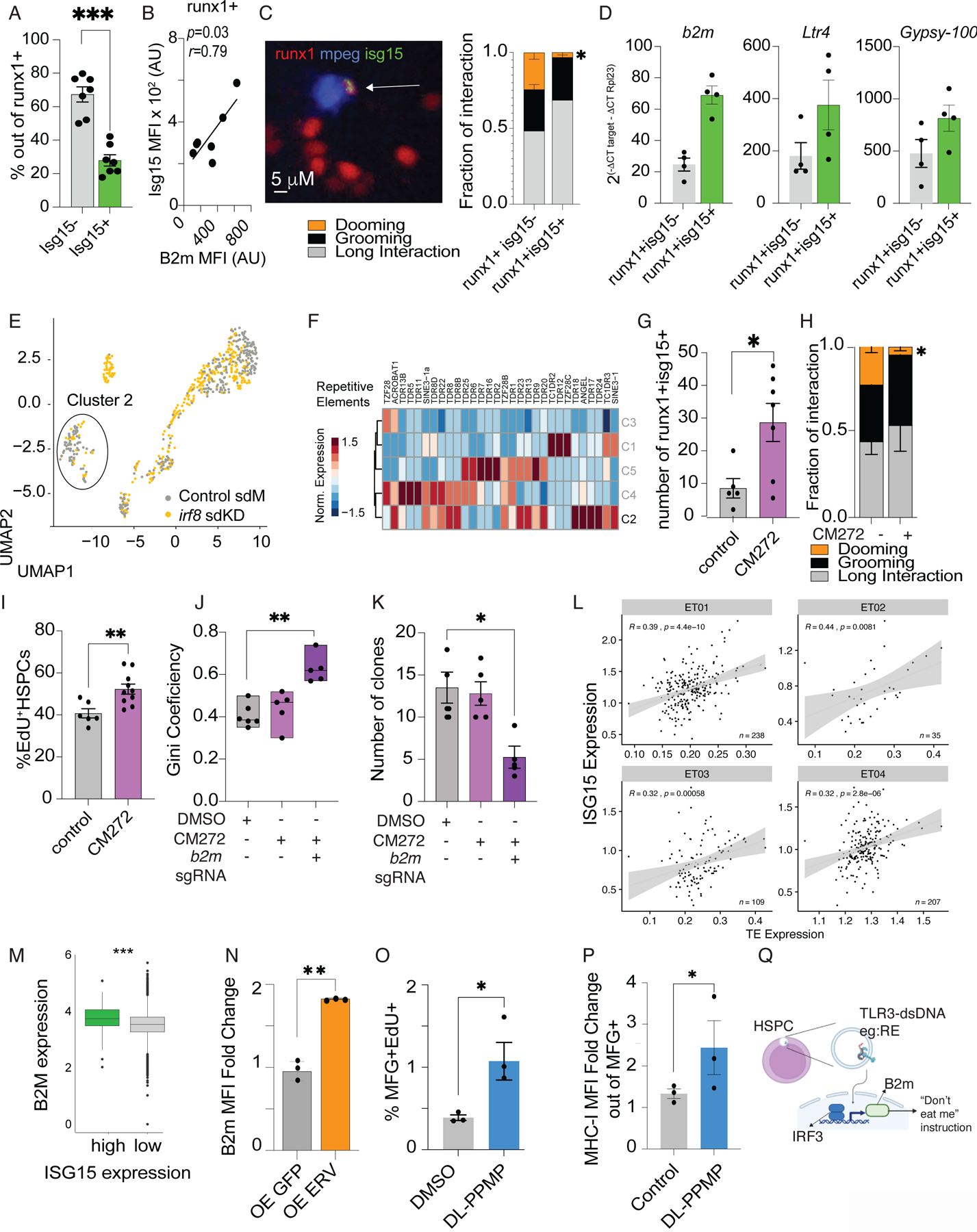
Repetitive elements as *Erv* elicit a virus-like response in HSPCs promoting B2m expression. (A) Flow cytometry shows that ~ 20% of *runx1+23mCherry+* HSPCs expresses isg15 (gating strategy is shown in fig. S4A). Data points represent a pool of ~200 embryos acquired in 3 independent experiments. Data are means ± SEM. Statistical analysis performed by Mann-Whitney test. *P* = 0.003. (B) Positive correlation between B2m and Isg15 levels. Data were analyzed by Pearson correlation. Data points represent a pool of ~200 embryos acquired in 3 independent experiments. (C) Left panel representative image of a Isg15+-HSPC interaction. HSPCs, red; macrophages, blue;isg15, green. Right panel: HSPCs-Isg15+ cells showed lower dooming ratios. (D) Sorted isg15+HSPCs showed higher *b2m* and RE expression. Data points represent a pool of ~200 embryos. Experiments were performed 2 times. Y-axis depicts the relative gene expression (E) Uniform manifold and projection (UMAP) of sorted *runx1+23-mcherry* HSPCs in standard morpholino (gray, Control sdM) and *irf8* depleted embryos (yellow, *irf8* sdKD) (Original data from (6)). (F) Cluster 2 (C2), enriched for control HSPCs, showed higher expression of RE. We analyzed 808 cells for this analysis. (G) Treating 2 dpf embryos with CM272 (5 uM) led to higher number of isg15+HSPCs in the 3dpf CHT. Data was quantified by live cell imaging Data were analyzed by Mann-Whitney test. *P=0.02. Data points represent an embryo. Experiments were performed 2 times. (H) EdU staining of r*unx1+23:mCherry* embryos treated with CM272 identifies a significant increase in proliferating HSPCs at 3dpf. Data were analyzed by Student’s t-test. Data normality was enquired by the Shapiro-Wilk test. **P=0.0061. Data are means ± SEM. Control n= 5 and CM272 n=7. (I) CM272 treated embryos showed lower dooming ratios. Data points represent an embryo. Percentage was calculated by quantification of Edu+ Runx1+ cells divided by the total number of runx1+ cells. The plot depicted 2 independent experiments. Control n=6 and CM272 n=10 (J) Gini coefficient in control (DMSO), CM272 and CM272 *b2m* knockdown zebrafish. Data were analyzed by Kruskall-Wallis test followed by Dunn’s multiple comparison test. **P=0.0043. Data points represent an adult fish. DMSO n=6, CM272 n=5 and b2m sgRNA n=5. The plot depicted 2 independent experiments.(K) *b2m*-TWISTR *Zebrabow-M;draculin:CreERT2* treated CM272 exhibits reduced numbers of HSC clones in the adult marrow. Data were analyzed by Kruskall-Wallis test followed by Dunn’s multiple comparison test. *P=0.023. Data points represent an adult fish. DMSO n=6, CM272 n=5 and b2m sgRNA n=5. The plot depicted 2 independent experiments. (L) *ISG15* expression positively correlates to the transposable elements (TE) expression in human HSCs. Previously published single cell RNA-seq datasets of human bone marrow CD34+ stem and progenitors (original data from (90), n = 4 samples) were aligned to a TE reference using CellRanger v3.2. TE expression was normalized by total RNA counts. Pearson correlation was performed between TE expression and *ISG15* gene expression of individual HSCs, represented in each datapoint. (M) Human bone marrow HSCs from the same dataset as in (L) were grouped based on their *ISG15* expression, where in the upper quartile was considered the threshold for high and the lower quartile as low. Normalized expression of *B2M* is shown for individual cells. *ISG15* high cells showed higher *B2M* expression. Only cells with at least one transcript of *ISG15* were included in the analysis. High, n = 203 cells, low n = 608 cells, from n = 4 samples; *P* = 0.02 using a linear mixed model, with sample as a random effects variable. Box plot represents the median, bottom and top quartiles, whiskers correspond to 1.5 × the interquartile range. (N) Human *Erv* overexpression upregulated the B2M levels on human CD34+ cells. Data are means ± SEM. Data points depict a CD34 donor. The data collected from 2 independent (O) EdU staining of MFG+ mice treated with DL-PPMP identifies an increase in proliferating HSPCs 24 hrs after the treatment. Percentage was determined by the total number of living cells. Data points depict a mouse. (P) DL-PPMP treatment stimulates B2M expression in murine HSPCs. Flow cytometry was performed using the cells from Tibia/Femur (gating fig. S4Q). Data were analyzed by Student’s t-test. *P=0.02. Data are means ± SEM. n mice = 3 per condition. (Q) A schematic overview of the proposed molecular pathway resulting in higher surface B2m.

Increased expression of *b2m* is a classic response against viral infection and a cellular response to type I IFN (37–39). To investigate the intrinsic heterogeneity of the HSPC population, we used the IFN-stimulated gene 15 (*isg15*) as a reporter for the Tlr3-mediated Irf3 response (37–39). Flow cytometry revealed that 27% of Runx1^+^ HSPCs were positive for *isg15* (Fig. 4A, (fig. S4A) and positively correlated with B2m surface presentation (Fig. 4B). Isg15^+^ HSPC were less likely to be doomed by macrophages (Fig. 4C, movie S6). The associated enrichment of *isg15* activity and B2m within select HSPCs hinted at a viral mimicry response in these cells. To test this, we injected 72 hpf embryos with a Tlr3 agonist, Poly I:C, and measured the fraction of Isg15+HSPCs. Poly I:C increased the fraction of Isg15+ HSPCs (fig. S4B). Collectively, these data suggest an endogenous, viral mimetic response could trigger the type I IFN pathway, leading to high B2m levels on the HSPCs surface, and consequently protecting the HSPCs from dooming.

Repetitive elements (REs), including endogenous retrovirus (*Erv*) have roles in regulating HSPC formation and regeneration. Therefore, we hypothesized that REs could be the endogenous Tlr3 ligand mediating *b2m* expression (40–42). qPCR analysis of Runx1^+^Isg15^+^ cells revealed a consistent increase in endogenous retroviral and B2m expression (Fig. 4D). Together, these data suggested that HSPCs with higher levels of REs transcripts also present elevated levels of B2m.

To gain insight into the REs status in macrophage-interacting HSPCs, we re-analyzed published scRNAseq data of sorted *runx1+23:mCherry* HSPCs from macrophage-depleted and control embryos (6), and modified the mapping to include the expression of REs, including long terminal repeats (LTRs), at the single-cell level (43). The cluster of cells enriched for cell cycle gene expression (Cluster 2) had elevated RE expression than the cluster enriched for non-cycling cells (Fig. 4E, 4F, fig. S4C, S4C). This suggested HSPCs that are primed to proliferate have higher RE expression. Additionally, bulk-RNAseq of Runx1^+^ cells from the CHT of embryos treated with DL-PPMP (pro-grooming, B2m-dependent compound), showed an upregulation of REs, including transcripts for *Ltrs* (fig. S4D).

To evaluate B2m expression in HSPCs carrying high or low content of *Erv* transcripts, we crossed a zebrafish reporter line for a zfERV (LTR5) (44) with *runx1+23:mCherry* zebrafish. LTR5^+^ HSPCs exhibited higher B2m levels by FACS (fig. S4E). We next sought to determine if RE levels were dependent on macrophage-HSPC interactions. To test this, we quantified the levels of cytosolic dsRNA in Runx1+ cells from wild-type or macrophage-depleted embryos at 48 hpf. Macrophage depletion did not change the dsRNA content in Runx1^+^ cells (fig. S4F), suggesting the endogenous content of REs in HSPCs is independent of macrophage interaction.

Overall, these data demonstrated that elevated cytosolic REs, including Ltrs, correlated with higher B2m levels, suggesting that a viral mimicry program could protect HSPCs from macrophage dooming.

To determine whether inducing the expression of RE in HSC could regulate macrophage behavior we treated 48 hpf zebrafish embryos with the G9a/DNMT inhibitor, CM272 (45). We reasoned that inhibiting DNA methylation would enhance RE expression, including *Erv* (46, 47). Indeed, after 24 hrs, HSPCs from embryos treated with CM272 increased expression of endogenous retrovirus and virus-like response genes (fig. S4G). Additionally, CM272 increased the proportion of Isg15^+^ and B2m^+^ HSPCs and increased the level of Calr on HSPCs (fig. S4H-S4K). This suggested that CM272 could promote HPSC-grooming by macrophages. We treated 72 hpf embryos with CM272. CM272 increased the number of Isg15+ HSPCs, HSPC proliferation, and reduced HSPC-dooming in live imaging experiments (Fig. 4G-I, movie S7-S8).

As CM272 might have the potential to regulate dooming and grooming via a number of gene expression changes beyond upregulation of REs, we cloned the zebrafish *ltr4* downstream the *runx1*+23 enhancer-GFP to promote the overexpression of a RE in the HSPCs. We found that HSPCs from embryos overexpressing *ltr4 (ltr4-OE)* showed higher proliferation compared to embryos overexpressing the *runx1*+23 enhancer-GFP (fig. S4L). Considering that macrophage grooming and longer interactions correlate with higher HSPC proliferation (6), the increased proliferation observed in the *ltr4-OE* may reflect higher grooming behavior. Thus, increasing global RE expression (by CM272) or overexpression of a specific RE, Ltr4, protected against macrophage dooming.

We investigated the potential consequences of RE-mediated protection from reduced dooming events on HSPC clonality. We opted to use CM272 treatment as a tool to increase the “don’t eat-me” signal on HSPCs, as it enabled us to focus on the events after HSPCs budding (which occurs at 24–48 hpf). We reasoned that the derepression of retroviral elements would increase the proliferation of HSPCs. Indeed, CHT imaging confirmed higher proliferation rates of HSPCs, as depicted by EdU incorporation (Fig 4H). However, zebrabow analysis showed virtually no difference in clonality (Fig. 4J-K), suggesting that CM272 promotes the amplification of already established clones. We did not observe a rescue in clonal dominance when we treated TWISTR-*b2m* mutants with CM272, confirming that dsRNA sensing occurs upstream of B2m presentation. (Fig. 4J-K).

Together, these data showed that REs positively correlated with B2m^+^ HSPCs and that upregulating endogenous retroviral levels could provide protection against HSPC dooming.

### B2M induction via endogenous retrovirus is conserved throughout evolution

The sequence of amino acids for B2m is highly conserved (48, 49). Thus, we examined whether ERV-driven B2M expression is conserved in mammals. Consistent with our observation in zebrafish, we found that human *ISG15*^+^ HSPCs (50) had higher *B2M* expression and positively correlated to the expression of REs (Fig. 4L-M).

We overexpressed human *Erv* or *GFP* (as control) in CD34 cells to validate a causal relationship between B2M levels and REs expression. Overexpression of *Erv*, but not control GFP, led to an increase in B2M on the surface of HSPCs (Fig. 4N, fig. S4M-S4N). In contrast, ROS levels were not elevated following *Erv* overexpression (fig. S4M). The surface B2m increase was observed in CM272-and Poly I:C-treated human CD34 cells, which could be abrogated by blocking Tlr3 signaling (fig. S4O). Our data from human cells and zebrafish suggested the regulation of B2M via dsRNA/Tlr3 signaling is conserved throughout evolution.

To understand the functional relevance of the “don’t eat-me” signal in the mammalian system, we treated myelodysplastic syndrome 1 (*Mds1*)-^*GFP+*^*/Flt3*^*Cre*^ (MFG) stem cell reporter mice (51) with DL-PPMP as it promoted the presentation of B2M, but not other “don’t eat-me” molecules, such as CD47 (fig. S4P). We chose this reporter model because *Mds1* is a gene highly enriched in long term HSCs (LT-HSCs) that are capable of self-renewing (52). We found that DL-PPMP treatment increased HSPC proliferation (Fig. 4O, fig. S4Q) and increased MHC-I levels (Fig. 4P, fig. S4Q). This suggested that the HSPCs were protected from the macrophage dooming and proliferated as a consequence of grooming.

### B2m expression in response to repetitive elements alters HSPCs fate

Endogenous retroviral proteins and genetic material have already been shown to regulate innate immune response (53, 54). ERV-derived enhancers and promoters appear to be activated upon pathogen infections, suggesting that for a cooperative activation in responding to pathogens (55, 56), or the existence of a trained immunity (57) function between innate immune response and ERVs.

Given the evolutionary conservation of RE and B2m, we then sought to investigate the relevance of this finding in the context of pathogen infection, which is an evolutionarily conserved process. We evaluated the role of Tlr3 signaling in mediating “emergency granulopoiesis”. This is a distinctive, protective, program of accelerated de novo production of neutrophils from amplification of progenitor cells in response to fatal infection (58, 59). Therefore, to explore if Poly I:C could induce emergency granulopoiesis we treated zebrafish embryos with Poly I:C and assessed the numbers of neutrophils as a proxy of myeloid emergency response. We found that upon Poly I:C stimulation the population of neutrophils increased (fig. S4R). Similarly, humanized NOG mice showed higher granulopoiesis and GM-CSF levels upon infection (60).

Although further studies are needed to strengthen the relevance of this phenomenon, our results suggested that the viral stimulation may confer a better fit against opportunistic pathogens by promoting the granulocyte differentiation.

Besides the pathogenic aspects, we also found higher *B2m* expression in AML-malignant HSPCs, and higher TE expression in human AML blast cells (fig. S5A-B). This suggested that AML cells might hijack the “don’t eat-me” signal to avoid macrophage elimination.

Taken together, our data demonstrate that viral-mimetic signaling of RE-Tlr3 mediates the levels surface B2m on HSPC to promote macrophage-mediated grooming and protect against macrophage-dooming in an evolutionary conserved manner that shapes HSCs fate determination and clonal proliferation (Fig. 4Q).

## Discussion

Our data support a model in which macrophages vet the quality of newly formed HSPCs through a balance between “eat-me” and “don’t eat-me” signals. This process is mediated by the inputs provided by surface Calr (“eat-me”) and B2m (“don’t eat-me”), which are driven by ROS and dsRNA-Tlr3 signaling, respectively. In our proposed model, surface Calr governs the macrophage-HSPC interaction. While B2m, a “don’t eat me” signal dictates macrophage’s grooming or dooming behavior.

B2m is an evolutionarily conserved molecule that positively responds to type I IFN (49, 61–65). The interferon family has been previously reported to break stem cell quiescence and promote asymmetric division (66, 67) and stimulate embryonic HSC maturation (68). Type I IFN regulates the Jak-Stat pathway, mediated by STAT1 phosphorylation, resulting in HSCs proliferation and activation. Our work expanded the implications of type I IFN as it shows that Tlr3/dsRNA cascade triggers an interferon (Irf3) response that stimulates the expression of B2m on HSPCs. B2m acts as a “don’t eat-me” signal thereby inhibiting macrophage dooming of HSPCs. We envision that our findings on influence of B2m extend beyond the hematopoietic system, impacting tumor-associated macrophages (TAMs) (22, 69–71) and anti-CD47 treatment efficacy in tumors with high MHC-I (22). As species like zebrafish that lack CD47 orthologs, our work suggests B2m as a primitive signal on stem cells for mediating the “don’t eat-me” signal in macrophages.

We uncovered the role of RNA repetitive elements (RE) in regulating stem cell clonality by triggering Tlr3, mimicking a virus response that will result in the expression of B2m, a “don’t eat-me” molecule. Other TLRs responses may mediate Calr externalization. For instance, viruses such as flaviviruses (ssRNA, TLR7/8) can promote the externalization of Calr, which, in turn, is recognized by natural killer (NK) cells (72). B2m, which curtails macrophage-mediated engulfment, could also attenuate NK cell activity. ERV activation may accelerate the differentiation of HSPCs to immune cells that would help fight infection, providing an evolutionary selection to maintain HSPCs that have endogenous ERV activation. Our experiments using the demethylating agent, CM272, demonstrate that derepression of RE triggers B2m and this is associated with clonal persistence. Patients with myelodysplasia and leukemia are often successfully treated with demethylating agents (73–76). It is possible that the therapeutic response is due to RE activation and survival of normal or mutant clones associated with adequate differentiation by the don’t eat-me signal.

In the development context, REs have been shown to increase expression of RE RNA during endothelial-to-hematopoietic (ETH) transition, where they mediate HSPC formation (40). isg15+ HSPCs could be responding to REs reminiscent of the ETH process. In this scenario, the RE-harboring HSPCs from ETH would respond to the IFN program enabling the expression of B2m and competition for marrow colonization. This protective mechanism may also operate in adulthood in response to environmental stress, such as during infections or in clonal stem cell disorders, as leukemia. Supporting this hypothesis, the transposable elements expression were used to accurately predict acute myeloid leukemia (AML) prognosis (77). B2m could confer protection of myelodysplastic or leukemic clones resulting in the establishment of the diseases. Manipulating the levels of “don’t eat-me” and “eat-me” signals may have important therapeutic implications for immune therapy by harnessing the macrophage selective removal of a mutated stem cell clone.

## Materials and Methods

### Animal models

Wildtype zebrafish AB, *casper* or *casper*-EKK (78), and transgenic lines, *runx1+23*:*mCherry* (11), *Zebrabow-M draculin:CreER*^*T2*^ (31, 79), *mpeg1:BFP* (6), *mpeg1:EGFP* (80), *ltr5-EGFP* (44), *isg15-EGFP* (39), *Calr3a knockout (from ZIRC), irf8 knockout.* For the embryonic experiments we used 3 dpf embryos, while the adult experiments were conducted in 4–6 months old fish. Both genders were used for the experiments.

Wildtype 8–12 weeks old male and female C57BL/6J mice (Jackson Labs stock #000664) were also used in this study. All mice were housed in the Animal Facility of Harvard University or at the Boston Childrens Hopital mouse house and all the experiments and protocols were performed in compliance with the institutional guidelines of Harvard University. The animals were kept under *ad libitum* food and water and euthanized by CO_2_ asphyxiation

All animals were housed at Faculty of Art and Science in Harvard university and handled according to approved Institutional Animal Care and Use Committee (IACUC) of Harvard University protocols #15-03-237 and #11-21-3.

### Chemical Screen

HEK293 cells transfected with the SPLIT-Turbo ID (9) constructs were treated with the Sigma Lopac^®^1280 chemical library and BIOMOL/ICCB bioactive. Chemical libraries were in a 384-well format and were diluted into the 384-well format using robots. The final chemical compound concentration was 5 μM, 2.5 μM and 0.625 μM, in a final treatment volume of 50 μl RPMI supplemented with 10% Fetal Bovine Serum, 1% Glutamine, 1% Pen/Strep and 100 μM Biotin. After 24 hrs, the cells were manually stained with Streptavidin (#405235, Biolegend), A647-zenon (ThermoFisher, #Z-25408) conjugated Calreticulin (ThermoFisher, #PA3-900) and Hoechst (InvitroGen, #H3570). Screening of inducers and in vivo was performed using a Zeiss Cell Discoverer 7 Live/High content equipped with the Prime 95B camera with control CO_2_ and temperature. Cell profiler objected to identification and the R package R sight was used to identify the chemical hits inducing surface Calreticulin. The wells treated with DMSO were used as control.

### Genome-Scale CRISPR-Cas9 Knockout Screening in Human Cells

The human GeCKO v2 one-plasmid system library was purchased from Addgene (cat. number: 52962) (13, 81). This one-plasmid system contains a lenti/Cas9-blast plasmid. The amplification of these plasmids and lentiviral production was performed following the protocol described by Shalem et al (13)

K562 cells were cultured until 80% confluence and transduced using a spintransfection, which 100 × 10^6^ cells were transferred to a 1.5 mL microtube containing 8 mg/ml polybrene (Millipore, #TR-1003-G) and the lentiviral library. The MOI used CRISPR library transduction was of 0.3 to limit multiple lentiviral integration. Then, samples were centrifuged at 1000g for 2h at 34°C. After infection the cells were resuspended in IMDM-10% FBS (Fetal bovine serum Premium, Cat. No #S11150H, Lot. No #F22100) and 1% Glutamine to remove the remaining polybrene and non-transfected virus. After 24 h recovery, puromycin (1 µg/mL) was added to the culture to select the cells carrying the constructs. The cells were kept for 5 days under these conditions.

After the puromycin selection the cells were centrifuged at 500g for 5 min and resuspended in IMDM-10% supplemented with DMSO (vehicle control) or DL-PPMP (5μM). The cells were stimulated for 24 hrs and 10^6^ were kept for baseline correction (Day 0). Then labeled with zenon-conjugated Calreticulin (A647). The Calr^-^ cells were sorted in the FACSAria cell sorter (BD Biosciences). Before FACS sorting, cells were treated with the zombieDye (Biolegend) for cell viability.

### Gecko library preparation

At the end of the screen, the library preparation was generated by deep sequencing of nested PCR amplicons (82). In brief, genomic DNA from Calr^-^ sorted cells was extracted with DNeasy blood and tissue kit (Qiagen, 69506). The next-generation sequencing of the amplified sgRNA library was described elsewhere (82). Amplification was performed using Phusion^®^ High-Fidelity PCR Master Mix with HF Buffer (NEB, M0530), with the following conditions: 95°C 5 min, [98°C 20 seconds, 60°C 15 seconds, 72°C 15 seconds] x 18 cycles, followed by 72°C 1 minutes for extension. Then, PCR amplicons were purified on AMPure beads (Beckman Coulter).

### Mageck Analysis

We analyzed the sgRNA read count data from the CRISPR screen experiments with MAGeCK package. The mageck count command was employed to preprocess and normalize the data for control and different treatment groups. Then mageck mle command to perform gene essentiality analysis based on the design matrix of treatment vs control, and taking into account for non-Targeting Control Guide. The MAGeCK output file lists the gene summary including ranking of enriched genes. Metascape (83) was used for pathway analysis.

### Microscopy and image analysis

Time-lapse microscopy was performed using a Yokogawa CSU-X1 spinning disk mounted on an inverted Nikon Eclipse Ti microscope equipped with dual Andor iXon EMCCD cameras and a climate controlled motorized x-y or with the Zeiss CR7 Live/High content equipped with the Prime 95B camera with control CO_2_ and temperature.. Animals were only included for imaging and analysis if expression of all transgenes could be identified. Images were acquired using NIS-Elements (Nikon) or Zen Blue software, blinded, and processed using Imaris (Bitplane). Specimens were mounted in 0.4% low melting agarose with tricaine (0.16 mg/ml) in glass bottom and covered with E3 media containing tricaine (0.16 mg/ml).

### Flow cytometry

For embryonic stainings 3 dpf tails were chopped with a razor blade in cold PBS and then incubated in Liberase (Roche) for 30 minutes at 37 °C before filtering the dissociated cells through a 40 μm filter and transferring to PBS-1% FBS solution.

To collect adult kidney marrow, adult zebrafish (4 to 6-months-old) were anesthetized with fresh tricaine (0.02%) in E3 fish water and dissected under a Leica MZ75 light microscope. The kidney marrow was transferred into 1.5 mL microtube containing cold PBS (Gibco) supplemented with 2% fetal bovine serum (FBS, Gemini Bio-Products) and 1 USP units/mL heparin (Sigma), and then mechanically dissociated by pipetting. After single cell suspension the sample was passed through a 40-μm nylon mesh 5–10 minutes before FACS acquisition and stained with the Zombie Dye (Biolegened) for cell viability detection.

The CellROX Deep Red (Invitrogen C10422), Annexin V-FITC (BD Biosciences), JC-1 (Biotium, #30001), Mitotracker CMX and zymosan staining were performed according to manufacturer instructions.

The following antibodies were used for flow cytometry: anti-human B2M (APC/Fire^™^750, Biolegend, clone 2M2, #A17082A), anti-mouse F480 (AF594, Biolegend, clone BM8, #123140), anti-human CD34 (PeCy7, BD Bioscience, clone 8G12, #348791), Lineage selection kit (Pacific Blue, Biolegend, CD3, clone 17A2; anti-mouse Ly-6G/Ly-6C, clone RB6-8C5; anti-mouse CD11b, clone M1/70; anti-mouse CD45R/B220, clone RA3-6B2; anti-mouse TER-119/Erythroid cells, #133310), anti-mouse CD117 (APC, clone 104D2, Biolegend, S18020A), Sca-1 (PeCy7, Biolegend, clone D7, #108113).

Flow cytometric analysis was performed on a BD FACSFortessa or BD FACS symphony.Data were analyzed with FlowJo software version 10.

### Embryos generation for the chemical screen validation

For the chemical screen validation, *Casper* fish were spawned in iSpawns for 15 min, embryos collected into embryo medium (E3) and cleaned using a water gradient for 48 hrs. At 2 dpf the viable embryos were sorted and kept at 25–30 embryos per well into 6-well plates and treated with the 22 Calr-inducers candidates.

### Zebrabow color labeling

At 24 hours post-fertilization (hpf), embryos were transferred to 6-well plates at a density of 25–35 embryos per well and treated with 15 μM 4-hydroxytamoxifen (4-OHT) for 3–5 hours in the dark at 28.5°C (31). Zebrabow analysis was conducted using the zbow software. Only zebrafish with greater than 75% recombination efficiency were processed.

### Drug treatment

Drugs were added to embryo E3 media in 6-well plates with 20–30 embryos per well at 48 hpf and incubated for 24 hours. Inducers (Data File S1) were added at a concentration of 50 μM. Diphenylene iodonium (DPI, Sigma) was added to embryos at a concentration of 100 μM. VAS2870 (Sigma) was added at a concentration of 20 μM. CM272 (Cayman Chemicals, #25948) was added at a concentration of 2 μM. Poly I:C (Sigma) and CU-CPT4a (Cayman Chemicals, #30951) at a concentration of 50 μM.

### Morpholino injections

Tlr3 Morpholino was selected from the *zfin* database (84) and purchased at GeneTools. It was resuspended to 300 μM in nuclease free water, heated to 65°C for 5 minutes, and kept at room temperature. Embryos were injected into the yolk at the 1 cell stage with 10 ng of morpholino. Morpholino sequences are listed in Data File S3.

### Liposome injection

Zebrafish embryos were dechorionated and anesthetized with tricaine (0.16 mg/ml) on flat agarose disks. Approximately 1.5 nanoliters of liposomes loaded with either clodronate or PBS.

### Zebrafish EdU Labeling

Embryonic circulation was injected at 3 dpf with 1 nanoliter of 500μM EdU. Embryos were kept at 4°C for 1 hour, fixed in 4% paraformaldehyde for 1 hour, permeabilized with 0.1% Triton for 20 minutes at room temperature, and labeled with Alexa Fluor 647 using the Click-iT reaction (Thermo Fisher) for 30 minutes according to manufacturer instructions. Embryos were washed with PBS+0.5% Triton and blocked for 1 hour in 10% Normal Goat Serum, 0.5% Bovine Serum Albumin, 0.5% Triton. Samples were incubated in Rat anti-mCherry Alexa Fluor 594 (Invitrogen M11240, 1:200, RRID:AB_2536614) for 1 hour at room temperature and washed 5 times with PBS+0.5% Triton.

### Double stranded RNA (dsRNA) staining

Embryos were dissociated in 0.5 mg/mL Liberase TM (Roche) solution for 30 min at 37 C, then dissociated by pipetting and resuspended in FACS buffer (PBS-1%FBS-1mM EDTA). Cells were then fixed in PFA 4% at 4°C and permeabilized in PBS-0.1 tween (PBS-T) for 30 min on ice. Cells were washed twice with FACS buffer and incubated overnight with dsRNA anti-mouse J2 mAb (Millipore, #MABE1134) at a concentration of 1/100. The next day, three washes were performed in the FACS buffer, following incubation with a secondary antibody (goat anti-mouse IgG1 AF488) 30 min at room temperature.

### CRISPR-Cas9 mutagenesis

Target selection for CRISPR/Cas9-mediated mutagenesis was performed using CHOPCHOP)(85). The selected sgRNA (Data File S3) having GC-content lower than 55%, self-complementarity=0,MM0=<1, MM1=<1, MM2=<1, MM3<1. The sgRNA templates were generated using the protocol described by (86) using the mMESSAGE mMACHINETM SP6 Transcription Kit (Invitrogen, AM1340). The gRNAs were validated using T7 endonuclease I assay in 72 hpf embryos.

The editing efficiency was further validated by deep sequencing of PCR amplicons. In brief, genomic DNA from zebrafish tissue was extracted with DNeasy blood and tissue kit (Qiagen, 69506). The CRISPR loci of the targeted genes were amplified using the primers above and barcoded with the Illumina NGS adaptor. Amplification was performed using Phusion^®^ High-Fidelity PCR Master Mix with HF Buffer (NEB, M0530), with the following conditions: 98°C 3 minutes, [98°C 10 seconds, T annealing 10 seconds, 72°C 10 seconds] x 35 cycles, 72°C 5 minutes. T annealing was 63°C]. Then, PCR amplicons were purified on PCR purification kit columns prior to sequencing.

### sgRNA and Cas9 injections

The gRNAs were resuspended to 1 ng/uL in nuclease free water, protein TrueCut Cas9 protein (ThermoFisher, #A36498) was added to the solution and kept at room temperature for 5 minutes. Embryos were injected into the 1 cell stage with 0.2 ng of gRNA.

### Mutagenesis analysis

For the analysis the sequencing reads were first trimmed for quality and aligned to the GRCz11/danRer11 assembly using Bowtie2 (87) with the --very-sensitive setting. Mutations were quantified by the R software CrispRVariants-version 1.20 (88)using a minimum read count of 20. The R version in this analysis was 4.1.0 (2021-05-18).

### Single-cell RNA-seq analysis

Repetitive elements sequences from *Danio rerio* were obtained from RepBase. Sequencing data from (6) were then aligned to the repetitive element library using Bowtie2 (87). Reads that are uniquely aligned to the RE were then identified by filtering for mapping qualities greater than 5, and the number of reads aligning to each RE was counted using count features. To identify differentially expressed REs, DESeq2 R package (43).

### mRNA synthesis and CD34 overexpression

For the overexpression of human *Erv* the respective cDNA was amplified from CD34 cells with PCR and cloned into pJET1.2/ blunt cloning vector (CloneJET PCR Cloning Kit, Thermoscientific, K1232). The constructs were linearized and used as templates for *in vitro* mRNA synthesis (T7 mMESSAGE mMACHINE kit, Ambion, AM1344). Cells were electroporated with the Lonza P3 primary solution using the DZ100 program. The *Erv* genomic location was retrieved from the *Dfam* database (89).

### RNA extraction

3 dpf embryos were lysed in TRIzol (Invitrogen, #15596026) by mechanical force. To isolate RNA, chloroform was added followed by extraction and precipitation of the aqueous phase using 5 μg of ribonuclease (RNase)–free glycogen and 0.25 ml of isopropanol. The supernatant was discarded and the pellet was washed twice in 80% ethanol and lastly dissolved in 10 μl of H2O.

### Reverse transcription quantitative polymerase chain reaction (RT-qPCR)

RNA (500 ng) was reversely transcribed into complementary DNA using Superscript VIlo RT (Thermo Fisher Scientific). RT-qPCR reactions were performed using SYBR Green Master Mix (Roche, #4309155); the mix contained 6.25 μl of SYBR, 0.75 μl of primers (forward and reverse, 300 nM), and 4.5 μl of H2O.

### Mouse cell culture

Mouse bone marrow derived macrophages (BMDMs) were generated from bone marrow precursors by standard M-CSF culture. The tibia and femora where marrow content was fluxed flushed with cold PBS. The bone marrow suspension was passed through a 40 µm filter and pelted in a centrifuge at 500× *g* for 5 min. Cells were counted and re-suspended at 2 × 10^6^ cells/ml with 10%-FBS, 1% PenStrep-IMDM and 20 ng/ml M-CSF (Peprotech). The cell suspension was plated on a 6-well plate and incubated at 37°C, 5% CO2 (day 0). On days 3, 5 ml of fresh medium containing 20 ng/ml M-CSF was added on top of the pre-existing medium. Cells were harvested with cold PBS supplemented with 1 mM EDTA at day 5 of differentiation.

For 3D co-culture BMDMs were seeded at a concentration of 1 × 10^5^ cells/ml with autologous sorted HSPCs (Lineage-Sca1+cKit+, LSK+) in 40% Matrigel in a 96-well glass bottom plate. After adding the cells into the Matrigel solution the plates were centrifuged for 3 min at 75× *g* and 4°C to bring the macrophages and HSPCs into the same focal plane for imaging. Plates were then incubated at 37°C for 30 min to ensure complete polymerization of the gel. Samples were then left at room temperature for 10 min before 200 µl of 10%-IMDM medium were added on top of the Matrigel. Each well was imaged in 10 min intervals for 2 hr using the Zeiss AiryScan confocal.

### Macrophage line RAW-274 chemotaxis

To analyze the -inducers-driven macrophage migration toward chemoattractant, LPS (50 ng/µL), we used under-agarose chemotaxis assays (12). In brief, agarose gels were cast into 35 × 10-mm tissue culture dishes (Corning). After gel polymerization, wells with a diameter of 4 mm were punched into the gel in ~3-mm distances using a cartoon template. 12 wells were punched in the agarose gel per dish: the left one was loaded with the -inducer treated Raw274 or LPS treated Raw274. The right well was loaded with the chemoattractant. The cells were previously labeled with CellTracker Red following the manufacturer recommendation.

### Human CD34+ RNAseq analysis

Human bone marrow single-cell RNA-seq data was downloaded from the Disco database (50). The cells annotated as HSCs were analyzed for the expression of ISG15. HSCs with expression of at least one UMI of ISG15 were divided into ISG15-low and ISG15-high (i.e. upper quartile of normalized ISG15 expression) cells and analyzed for B2M expression. The gene expressions were normalized by total UMI per cell, multiplied by a scale factor of 10,000 and log transformed. Linear mixed effects analysis was performed using the lme4 package (v.1.2-1). ISG15 status was entered as the fixed effect and subjects as random effects. P-values were obtained by likelihood ratio tests of the full model with the fixed effect against the model without the fixed effect. For the correlation of ISG15 expression and TE transcripts, single cell RNA-seq fastq files of bone marrow CD34+ cells from individuals with *Calr*-mutated essential thrombocythemia (90) were processed using Cell Ranger (v. 7.1.0) using chm13v2.0.fa and T2T_CHM13_v2_rmsk_TE.gtf files from T2T-CHM13v2.0 (91) to annotate the aligned reads for TE. HSCs with at least one transcript expression of ISG15 were included to determine the correlation of ISG15 expression versus TE expression (normalized by total UMI per cell; Pearson’s correlation).

### In vivo Edu staining

Mice were retro-orbitally injected with 1x EdU (500μM) and DL-PPMP (0.8 mg/kg, Cayman Chemicals, #17236) or DMSO. After 24 hrs they were anesthetized by vaporized isoflurane (3–4% for induction and 1–2% for maintenance) and sacrificed by cervical dislocation. The bone marrow was isolated from pooled femora and tibia by flushing them with cold phosphate-buffered saline (PBS) containing 2% FCS. Lysis of erythrocytes was performed using ACK Lysing Buffer and the remaining cells were stained with zombie dye for viability, anti-Edu as described above anti-B2M (APC-Fire700, 2M2). Data were aquiredAria FACS Fusion II cytometer (BD). List of antibody panel repertoires are provided in Supplementary Data.

### Statistical analysis

Graphs and statistical analyses were done with Prism (Graphpad) and RStudio. For all graphs, error bars indicate mean +/− standard error (SEM). *P* values were obtained with two-tailed Student’s *t-*test, Mann Whitney U-test or One-way ANOVAs for all analyses as indicated. Sample sizes were chosen based on sample availability and power calculations determined from preliminary observations to detect a change of at least 33% with an α of 0.05 and a β of 0.8. For all experiments except Zebrabow color labeling, a randomized set of embryos from a mixture of clutches was split into control and perturbation conditions. All experiments were repeated at least once.

## Supplementary Material

Data file S2

Data file S3

Data file S1

MDAR checklist

Movie S1

Movie S3

Movie S2

Movie S4

Movie S6

Movie S5

Movie S8

Movie S7

Fig S1

Fig S2

Fig S3

Fig S4

Fig S5

18

## Data Availability

All data are available in the main text or the supplementary materials. There are no restrictions on sharing reagents used in the study. The RNAseq data were deposited at Sequence Read Archive (SRA) submission numbers: SRR28470429, SRR28470426, SRR28470427, SRR28470424, SRR28470428, SRR28470425 (PRJNA1092543).
