# Supplementary material for: Transcripts of repetitive DNA elements signal to block phagocytosis of hematopoietic stem cells
Source: Science. Author manuscript; Available in PMC 2025 Apr 22. (PMC12012832; doi:10.1126/science.adn1629)
Supplement: MDAR checklist [file NIHMS2062621-supplement-MDAR_checklist.pdf]

Materials Design Analysis Reporting (MDAR)

Checklist for Authors

The MDAR framework establishes a minimum set of requirements in transparent reporting applicable to studies in the life sciences (see Statement of Task: doi:10.31222/osf.io/9sm4x.). The MDAR checklist is a tool for authors, editors, and others seeking to adopt the MDAR framework for transparent reporting in manuscripts and other outputs. Please refer to the MDAR Elaboration Document for additional context for the MDAR framework.

For all that apply, please note where in the manuscript the required information is provided.

Materials:

**Newly created materials**	**indicate where provided: page no/section/legend**	**n/a**
The manuscript includes a dedicated "materials availability statement" providing transparent disclosure about availability of newly created materials including details on how materials can be accessed and describing any restrictions on access.	The manuscript includes a dedicated materials availability statement, on page 28 under the Data and materials availability section	

**Antibodies**	**indicate where provided: page no/section/legend**	**n/a**
For commercial reagents, provide supplier name, catalogue number and RRID, if available.	Streptavidin (#405235, Biolegend) A647-zenon (ThermoFisher, #Z-25408), Calreticulin (ThermoFisher, #PA3-900),Hoechst (InvitroGen, #H3570), Addgene (cat. number: 52962), (Fetal bovine serum Premium, Cat. No #S11150H, Lot. No #F22100), Neasy blood and tissue kit (Qiagen, 69506), Phusion® High-Fidelity PCR Master Mix with HF Buffer (NEB, M0530), AMPure beads (Beckman Coulter), Liberase (Roche), Zombie Dye (Biolegened), CellROX Deep Red (Invitrogen C10422), Annexin V-FITC (BD Biosciences), JC-1 (Biotium, #30001), Mitotracker CMX (#M7512), zymosan (Thermo Fisher, #P35364), anti-human B2M (APC/Fire™750, Biolegend, clone 2M2, #A17082A), anti-mouse F480 (AF594, Biolegend, clone BM8, #123140), anti-human CD34 (PeCy7, BD Bioscience, clone 8G12, #348791), Lineage selection kit (Pacific Blue, Biolegend, CD3, clone 17A2; anti-mouse Ly-6G/Ly-6C, clone RB6-8C5; anti-mouse CD11b, clone M1/70; anti-mouse CD45R/B220, clone RA3-6B2; anti-mouse TER-119/Erythroid cells, #133310), anti-mouse CD117 (APC, clone 104D2, Biolegend, S18020A), Sca-1 (PeCy7, Biolegend, clone D7, #108113), Diphenylene iodonium (DPI, Sigma), VAS2870 (Sigma), CM272 (Cayman Chemicals, #25948), Poly I:C (Sigma), CU-CPT4a (Cayman Chemicals, #30951), dsRNA anti-mouse J2 mAb (Millipore, #MABE1134), TrueCut Cas9 protein (ThermoFisher, #A36498), pJET1.2/ blunt cloning vector (CloneJET PCR Cloning Kit, Thermoscientific, K1232), mRNA synthesis (T7 mMESSAGE mMACHINE kit, Ambion, AM1344), TRIzol (Invitrogen, #15596026), Superscript VIlo RT (Thermo Fisher Scientific). The chemicals used for the screen can be found at Data Supplementary 2 and were purchased by the Cayman Chemicals.	

**DNA and RNA sequences**	**indicate where provided: page no/section/legend**	**n/a**
**Short novel DNA or RNA including primers, probes:** Sequences should be included or deposited in a public repository.	***Split-Turbo ID Construct***<br>C1_Calr_F<br>C2_Calr_R<br>C3_TurboID_C1<br>C4_TurboID_C2<br>C5_IsertC1-2<br>C6_IsertC1-2<br>C10_CDH2<br>C11_CDH2<br>C12_TurboID_C_C10<br>C13_TurboID_C_C10<br>C14_TurboID_C_C10<br>C15_TurboID_C_C10<br><br>***Crispants sgRNA***	

ATGCTGCTATCC[illegible]
CTCGTCCTTGGC[illegible]
ATAAGCTAGCG[illegible]
TGGTTCTATCTC[illegible]
TGGTTCTATCTC[illegible]
TTAGCGCTAGCT[illegible]
ATGAGCTGGGA[illegible]
TGCAGCATAAA[illegible]
GCTGTAGATCTT[illegible]
ATGGGCAGCTC[illegible]
CAACGAGCTGC[illegible]
GCTAAGATCTA[illegible]

	b2m_CRISPR_SP6	CCTCCATACGAT
	b2m_F	CATCTTCGCAAT
	b2m_R	TCAGCTAAGGTA
	tl3_CRISPR_SP6	CCTCCATACGAT
	tlr3_F	TCAGCTAAGGTA
	tlr3_R	AACTCAGAGAA
	irf3_CRISPR_SP6	CCTCCATACGAT
	irf3_F	TCAGCTAAGGTA
	irf3_R	GGCTATTCTAAC
	dessertRegion_CRISPR_SP6	CCTCCATACGAT
	calr3b_CRISPR_SP6	CCTCCATACGAT
	calr3b_F	TCAGCTAATTGT
	calr3b_R	ACTTTGTAGATG
	Illumina barcode	
	**Morpholino**	
	MO1-tlr3	5' - GTAAAAACA
	RT-qPCR oligos	
	Zebrafish_Gypsy100-LTR_DR#LTR/Gypsy_F	TGTAACCCAGAT
	Zebrafish_Gypsy100-LTR_DR#LTR/Gypsy_R	TGTAACACTACC
	Zebrafish_Gypsy101B-LTR_DR#LTR/Gypsy_R	TGTCACCGGTTC
	Zebrafish_Gypsy101B-LTR_DR#LTR/Gypsy_F	TGTAATGATGAC
	Zebrafish_Gypsy101-LTR_DR#LTR/Gypsy_F	TGTCACCGACTC
	Zebrafish_Gypsy101-LTR_DR#LTR/Gypsy_R	TGTAACGGGGA
	Zebrafish_LTR4_DR#LTR/Pao_F	TGTAGGAGCCA
	Zebrafish_LTR4_DR#LTR/Pao_R	TGTAGCGGCTTT
	Zebrafish_BEL10-LTR_DR#LTR/Pao_F	TGTAGAAAATCT
	Zebrafish_BEL10-LTR_DR#LTR/Pao_R	TGTAAAAAAATT
	Zebrafish_ERV1-10_DR-LTR#LTR/ERV1_F	TGAGAGAACAT
	Zebrafish_ERV1-10_DR-LTR#LTR/ERV1_R	TGTAAGATTTCC
	Zebrafish_ERV1-10_DR-LTR#LTR/ERV1_F	TGAGGTGGATT
	Zebrafish_ERV1-10_DR-LTR#LTR/ERV1_R	TGTGGTGGTTTT
	Zebrafish_SINE3-1a#SINE/5s_F	CAGCTAGCTCTC
	Zebrafish_SINE3-1a#SINE/5s_R	TATATAGCGCTT
	Zebrafish_SINE3-2_DR#SINE/5s_F	GGACCAGGGTG
	Zebrafish_SINE3-2_DR#SINE/5s_R	TACTTCCCAGGT
	Zebrafish_ifnphi_F	CAAGCCGCTTGT
	Zebrafish_ifnphi_R	CATTCTTTGAGG
	Zebrafish_mx1a_F	AGACCAAAGCTC
	Zebrafish_mx1a_R	CATCTCATCTTG
	Zebrafish_mx1b_F	GCTGCATCTCGA
	Zebrafish_mx1b_R	CGGCTCAGTAA
	Zebrafish_isg15_F	CAGTACTTCAAC
	Zebrafish_isg15_R	AGTTTTGACCTG

	Zebrafish_Calr3a_F	CCGTAGTCGGA [illegible]
	Zebrafish_Calr3a_R	TTTATCCAGTCG [illegible]
	Zebrafish_Calr3b_F	GTCCGCCTGGTT [illegible]
	Zebrafish_Calr3b_R	TGATATCTGTGG [illegible]
	Zebrafish_rpl23_F	GGCATTGAATAT [illegible]
	Zebrafish_rpl23_R	ATTGTGAAAGC [illegible]
	Zebrafish_b2m_F	CCACTCCGAAA [illegible]
	Zebrafish_b2m_R	ATTCATCTCCTT [illegible]
	Human guiges	
	TLR3_hs_crRNA	ACATTAGATCTG [illegible]
	XBP1_hs_crRNA	TGGCCTTGTAGT [illegible]
	DDX3X_hs_crRNA	TTTGGCAAATTT [illegible]
	Human cloning	
	Human_DF0000471.4 LTR26C_F	TGAAGCCGTCCT [illegible]
	Human_DF0000471.4 LTR26C_R	TGTAACCGTAAT [illegible]
	Zebrafish cloning	
	Zebrafish_LTR4_DR#LTR/Pao_F	TGTAGGAGCCA [illegible]
	Zebrafish_LTR4_DR#LTR/Pao_R	TGTAGCGGCTTT [illegible]
	Zebrafish_ERV1-10_DR-LTR#LTR/ERV1_F	TGAGAGAACAT [illegible]
	Zebrafish_ERV1-10_DR-LTR#LTR/ERV1_R	TGTAAGATTTCC [illegible]
	The RNAseq data were deposited at Sequence Read Archive (SRA) submission numbers: SRR28470429, SRR28470426, SRR28470427, SRR28470424, SRR28470428, SRR28470425 (PRJNA1092543).	

Cell materials	indicate where provided: page no/section/legend	n/a
**Cell lines:** Provide species information, strain. Provide accession number in repository **OR** supplier name, catalog number, clone number, **OR** RRID.	HEK293 - RRID:CVCL_0045<br>K562 - RRID:CVCL_UC15<br>CD34 – Fred Hutch Cancer Center	
**Primary cultures:** Provide species, strain, sex of origin, genetic modification status.	CD34 – Fred Hutch Cancer Center<br>Male<br>Carrying no genetic modification	

Experimental animals	indicate where provided: page no/section/legend)	n/a
**Laboratory animals or Model organisms:** Provide species, strain, sex, age, genetic modification status. Provide accession number in repository **OR** supplier name, catalog number, clone number, **OR** RRID.	Wildtype zebrafish AB, *casper* or *casper*-EKK, and transgenic lines, *runx1+23:mCherry, Zebrabow-M draculin:CreER^{T2}, mpeg1:BFF, mpeg1:EGF, ltr5-EGFP, isg15-EGFP, Calr3a knockout (from ZIRC), irf8 knockout.* For the embryonic experiments we used 3 dpf embryos, while the adult experiments were conducted in 4-6 months old fish. Both genders were used for the experiments.<br><br>Wildtype 8-12 weeks old male and female C57BL/6J mice (Jackson Labs stock #000664) were also used in this study. All mice were housed in the Animal Facility of Harvard University or at the Boston Childrens Hopital mouse house and all the experiments and protocols were performed in compliance with the institutional guidelines of Harvard University. The animals were kept under *ad libitum* food and water and euthanized by CO_2 asphyxiation<br><br>All animals were housed at Faculty of Art and Science in Harvard university and handled according to approved Institutional	

	Animal Care and Use Committee (IACUC) of Harvard University protocols #15-03-237 and #11-21-3.	
**Animal observed in or captured from the field:** Provide species, sex, and age where possible.		n/a

Plants and microbes	indicate where provided: page no/section/legend)	n/a
**Plants:** provide species and strain, ecotype and cultivar where relevant, unique accession number if available, and source (including location for collected wild specimens).		n/a
**Microbes:** provide species and strain, unique accession number if available, and source.		n/a

Human research participants	indicate where provided: page no/section/legend) or state if these demographics were not collected	n/a
If collected and within the bounds of privacy constraints report on age, sex and gender or ethnicity for all study participants.		n/a

Design:

**Study protocol**	**indicate where provided: page no/section/legend)**	**n/a**
If study protocol has been pre-registered, provide DOI. For clinical trials, provide the trial registration number **OR** cite DOI.		n/a

**Laboratory protocol**	**indicate where provided: page no/section/legend)**	**n/a**
Provide DOI **OR** other citation details if detailed step-by-step protocols are available.		n/a

**Experimental study design (statistics details)**		
**For in vivo studies:** State whether and how the following have been done	**indicate where provided: page no/section/legend. If it could have been done, but was not, write not done**	**n/a**
Sample size determination	Use if gPower calculation	
Randomisation	yes	
Blinding	yes	
Inclusion/exclusion criteria		n/a

**Sample definition and in-laboratory replication**	**indicate where provided: page no/section/legend**	**n/a**
State number of times the experiment was replicated in laboratory.	The exact number of timed that the experiment was replicated in laboratory is indicated in the figure legends; However, the assay were performed at least 2 times	
Define whether data describe technical or biological replicates.	The cell lines experiments were performed at leats 2 times having 3 technical replicates. The animal experiments were conducted having biological replicates indicated in the figures legends and shown as dots in the figures.	

**Ethics**	**indicate where provided: page no/section/legend**	**n/a**
**Studies involving human participants:** State details of authority granting ethics approval (IRB or equivalent committee(s), provide reference number for approval.		n/a
**Studies involving experimental animals:** State details of authority granting ethics approval (IRB or equivalent committee(s), provide reference number for approval.	Approved Institutional Animal Care and Use Committee (IACUC) of Harvard University protocols #15-03-237 and #11-21-3.	
**Studies involving specimen and field samples:** State if relevant permits obtained, provide details of authority approving study; if none were required, explain why.		n/a

**Dual Use Research of Concern (DURC)**	**indicate where provided: page no/section/legend**	**n/a**
If study is subject to dual use research of concern regulations, state the authority granting approval and reference number for the regulatory approval.		n/a

Analysis:

Attrition	indicate where provided: page no/section/legend	n/a
Describe whether exclusion criteria were preestablished. Report if sample or data points were omitted from analysis. If yes report if this was due to attrition or intentional exclusion and provide justification.		n/a

Statistics	indicate where provided: page no/section/legend	n/a
Describe statistical tests used and justify choice of tests.	indicated in the figure legend	

Data availability	indicate where provided: page no/section/legend	n/a
For newly created and reused datasets, the manuscript includes a data availability statement that provides details for access or notes restrictions on access.	Newly created: SRA, PRJNA1092543<br>Reused datasets:<br>- Wattrus S. et. al. 2022 : GSE196551<br>- vanGalen P. et. al. 2019 : GSE116256<br>- Lamb, J. et al, 2006: GSE5258<br>- Nam. A,S et al 2019: GSE117826	
If newly created datasets are publicly available, provide accession number in repository **OR** DOI **OR** URL and licensing details where available.	SRA, PRJNA1092543	
If reused data is publicly available provide accession number in repository **OR** DOI **OR** URL, **OR** citation.	- Wattrus S. et. al. 2022 : GSE196551<br>- vanGalen P. et. al. 2019 : GSE116256<br>- Lamb, J. et al, 2006: GSE5258<br>- Nam. A,S et al 2019: GSE117826	

Code availability	indicate where provided: page no/section/legend	n/a
For all newly generated custom computer code/software/mathematical algorithm or re-used code essential for replicating the main findings of the study, the manuscript includes a data availability statement that provides details for access or notes restrictions.		n/a
If newly generated code is publicly available, provide accession number in repository, **OR** DOI **OR** URL and licensing details where available. State any restrictions on code availability or accessibility.		n/a
If reused code is publicly available provide accession number in repository **OR** DOI **OR** URL, **OR** citation.		n/a

Reporting

MDAR framework recommends adoption of discipline-specific guidelines, established and endorsed through community initiatives. Journals have their own policy about requiring specific guidelines and recommendations to complement MDAR.

**Adherence to community standards**	**indicate where provided: page no/section/legend**	**n/a**
State if relevant guidelines (e.g., ICMJE, MIBBI, ARRIVE) have been followed, and whether a checklist (e.g., CONSORT, PRISMA, ARRIVE) is provided with the manuscript.	ARRIVE	
