# Supplementary figures and images for: Transcripts of repetitive DNA elements signal to block phagocytosis of hematopoietic stem cells
Source: Science. Author manuscript; Available in PMC 2025 Apr 22. (PMC12012832; doi:10.1126/science.adn1629)

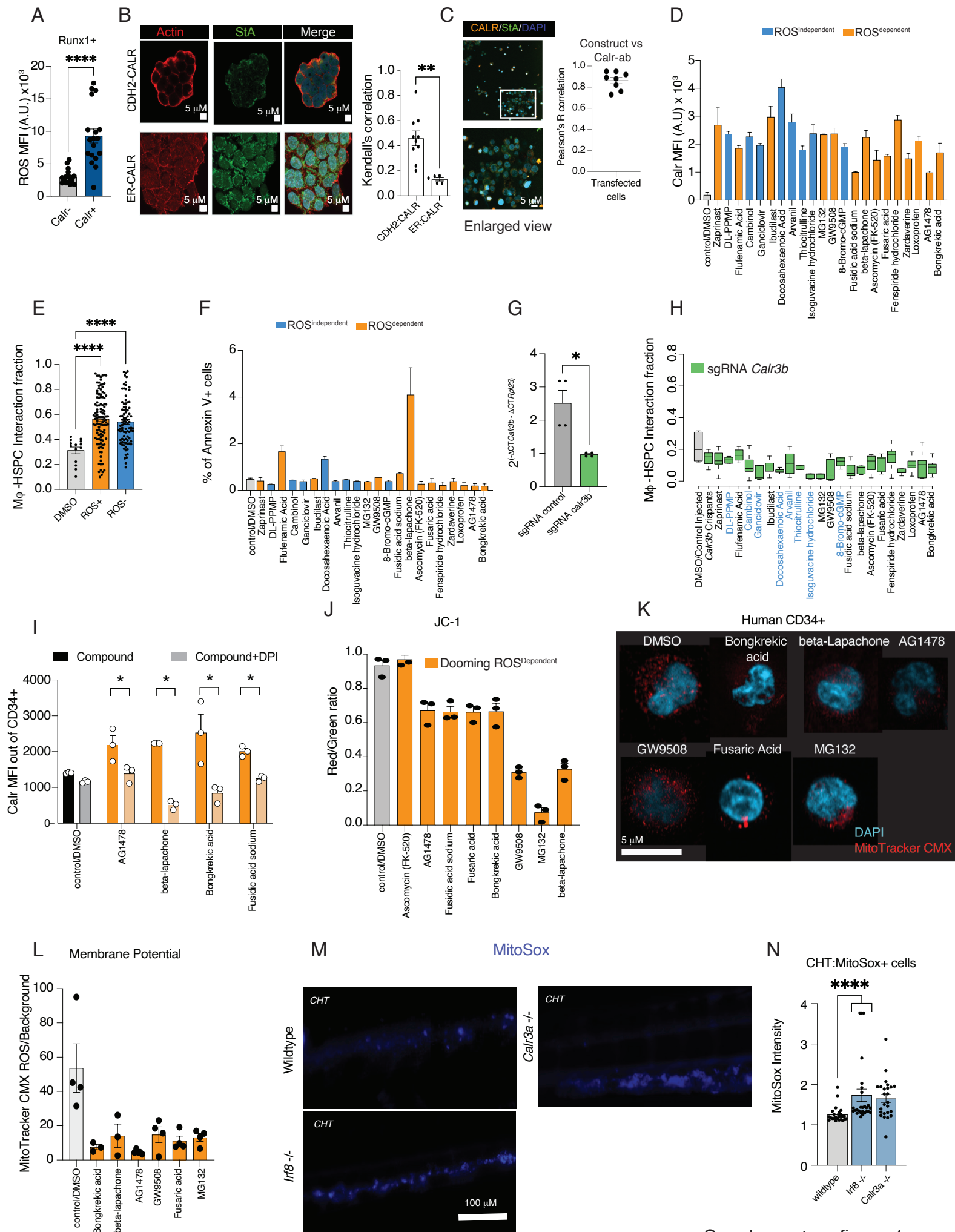

Supplementary figure 1

Supplement: Fig S1 [file NIHMS2062621-supplement-Fig_S1.pdf]

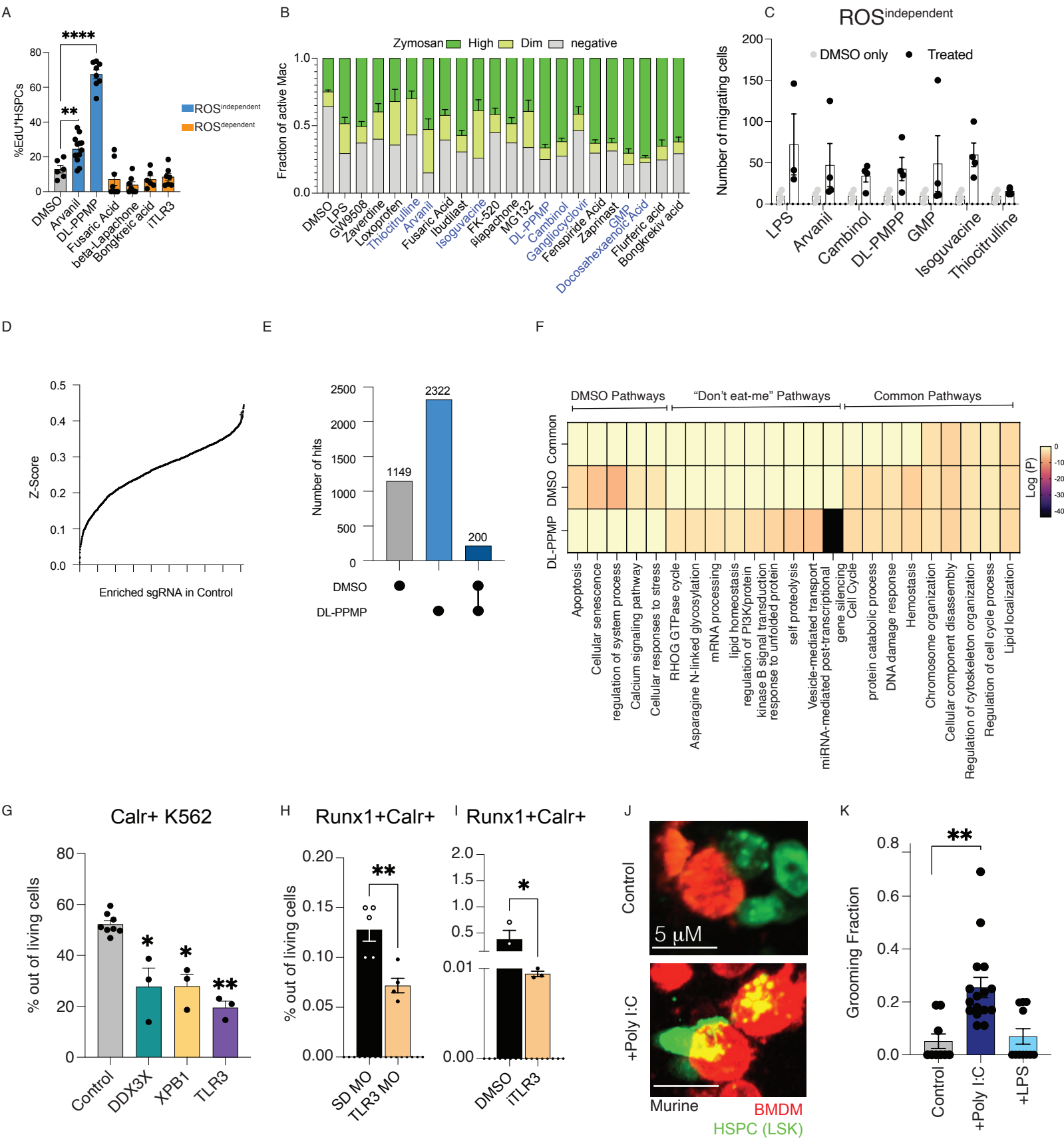

Supplementary figure 2

Supplement: Fig S2 [file NIHMS2062621-supplement-Fig_S2.pdf]

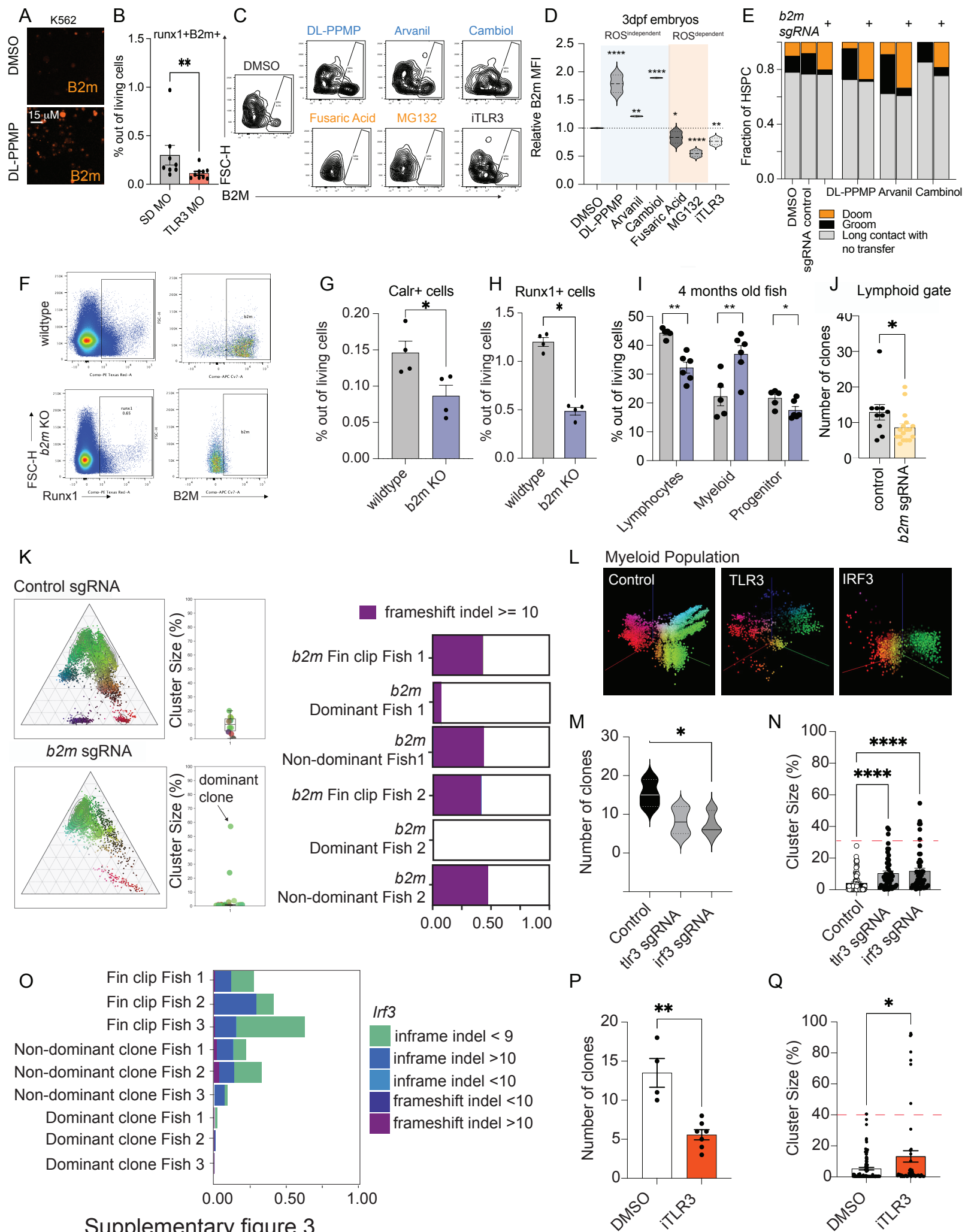

Supplementary figure 3

Supplement: Fig S3 [file NIHMS2062621-supplement-Fig_S3.pdf]

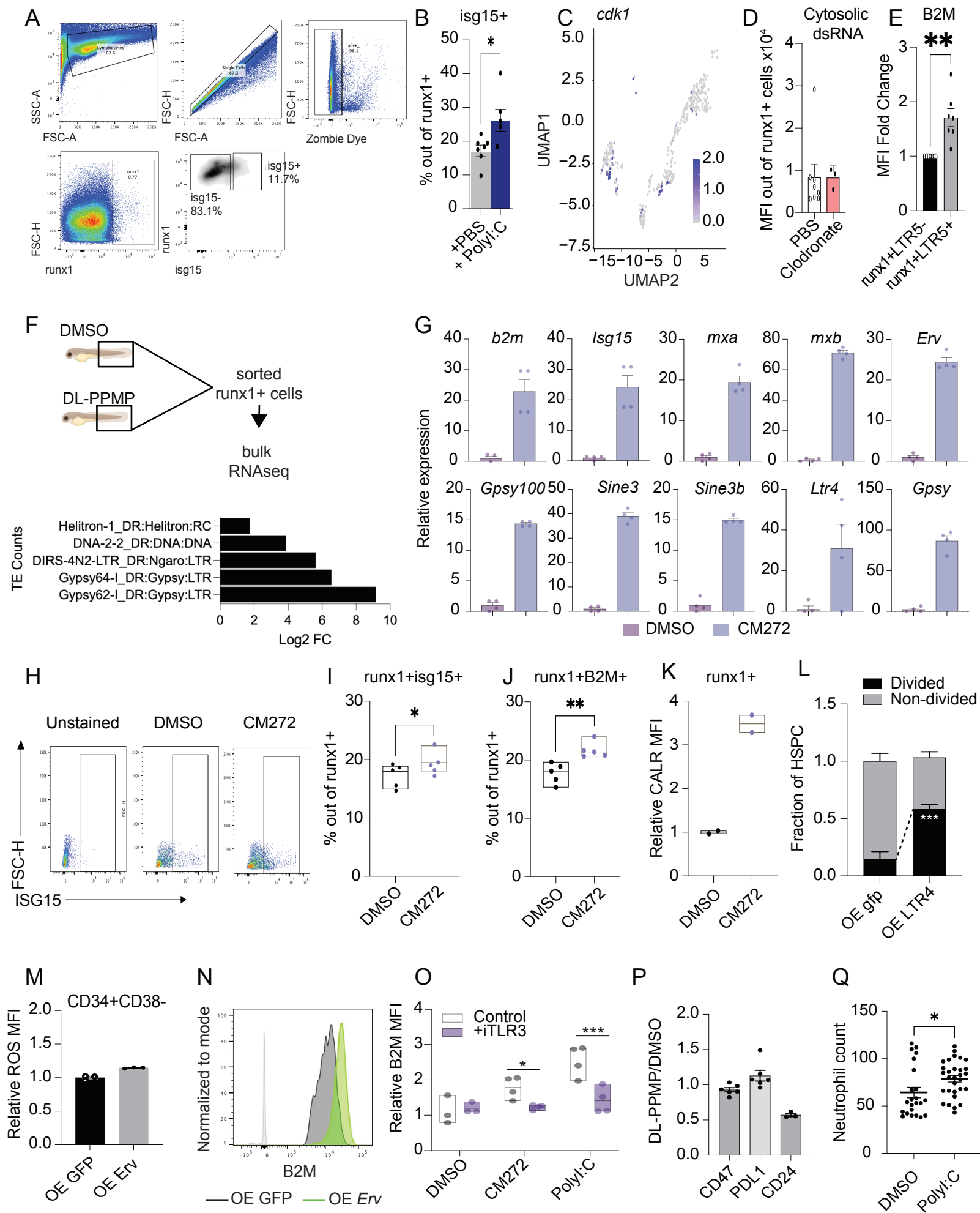

Supplementary figure 4

Supplement: Fig S4 [file NIHMS2062621-supplement-Fig_S4.pdf]

A

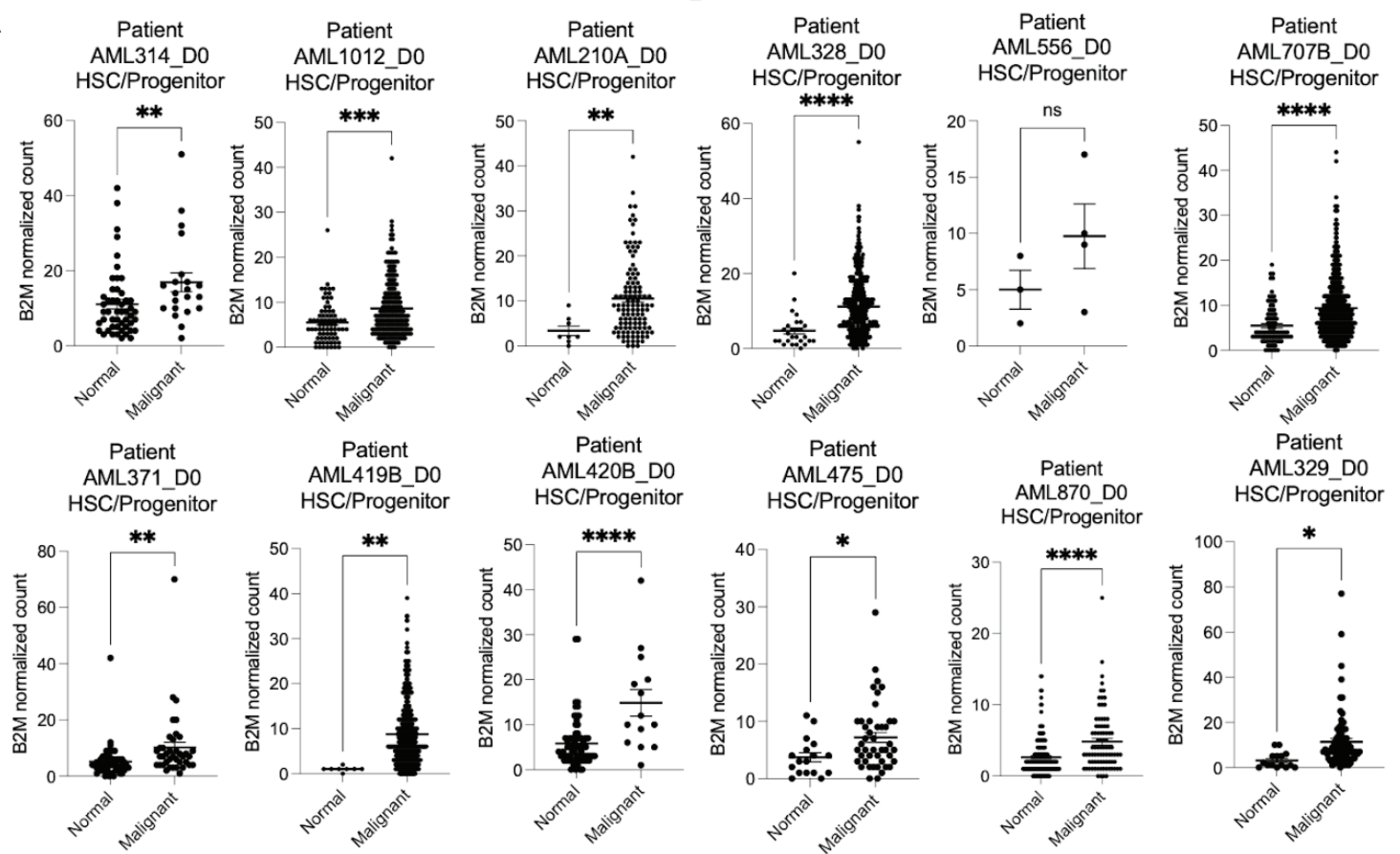

B

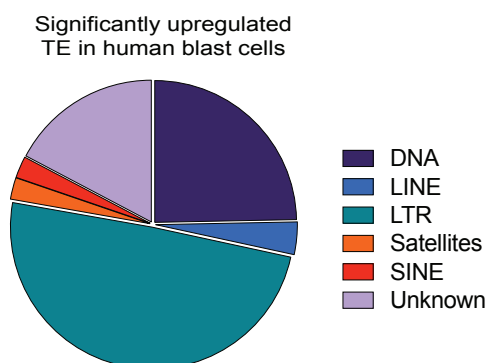

Supplement: Fig S5 [file NIHMS2062621-supplement-Fig_S5.pdf]
