# Supplementary material for: Transcripts of repetitive DNA elements signal to block phagocytosis of hematopoietic stem cells
Source: Science. Author manuscript; Available in PMC 2025 Apr 22. (PMC12012832; doi:10.1126/science.adn1629)
Supplement: 18 [file NIHMS2062621-supplement-18.pdf]

Science
AAAS

Supplementary Materials for

Transcripts of repetitive DNA elements signal to block phagocytosis of hematopoietic stem cells

Cecilia Pessoa Rodrigues[1,2], Joseph M. Collins[1,2], Song Yang[1], Catherine Martinez[2], Ji Wook Kim[1,2], Chhiring Lama[3], Anna S. Nam[3], Clemens Alt[4], Charles Lin[4], and Leonard I. Zon[1,2*]

Corresponding author: zon@enders.tch.harvard.edu

The PDF file includes:

Figs. S1 to S5 and their legends

Other Supplementary Materials for this manuscript include the following:

Movies S1 to S8
Data File S1 to S3

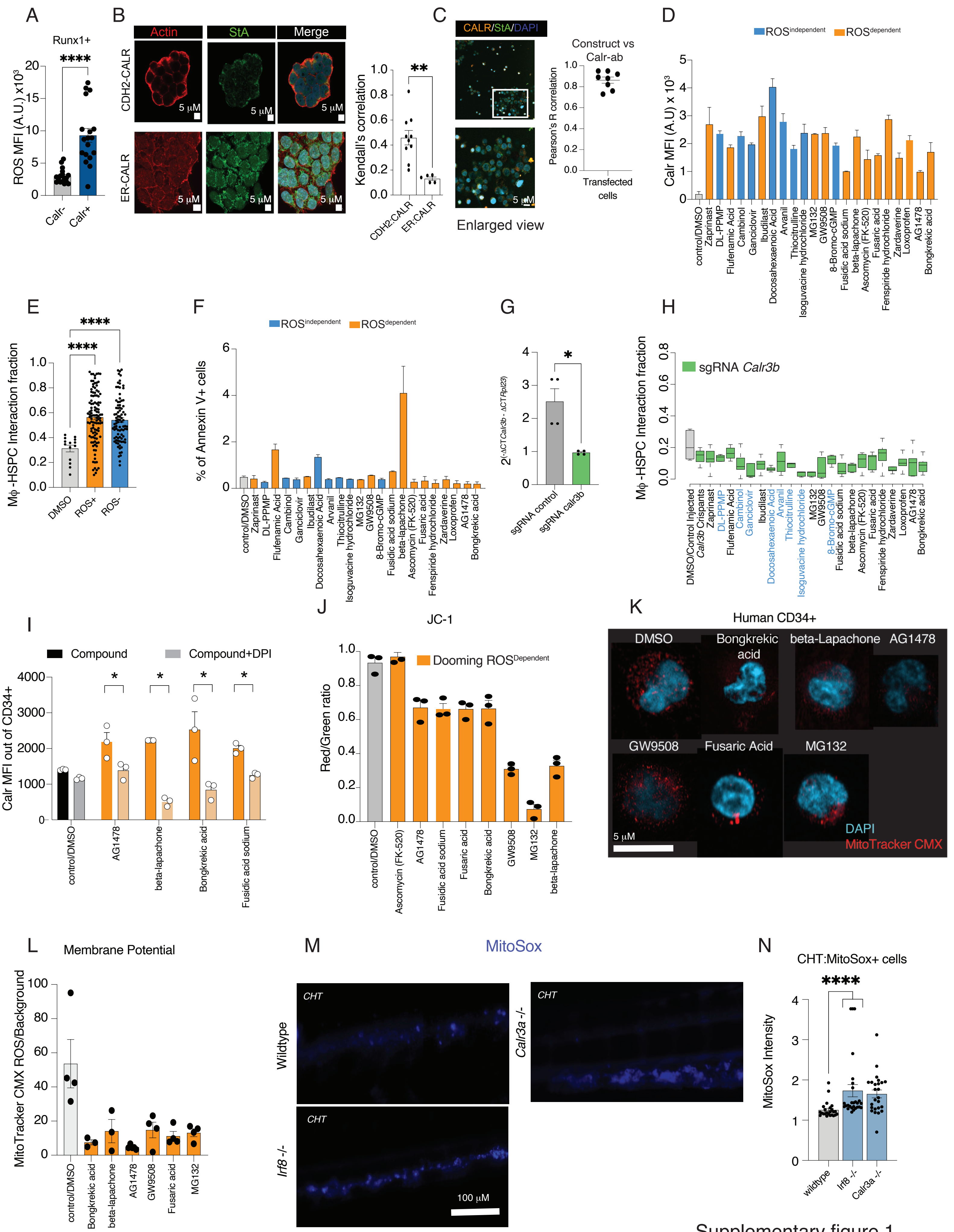

Supplementary figure 1

Fig S1. Identification of surface Calreticulin inducers. (A) Embryonic HSPCs marked by surface Calreticulin (Calr) exhibit higher levels of ROS. Data were analyzed by. MFI, median fluorescence intensity. A.U, arbitrary unit.unpaired Mann-Whitney; ****P<0.0001 (B) Representative image of the SPLIT-Turbo ID construct showing the CDH2-CALR (upper panel, membrane construct) and the Expresses split-TurboID C-terminal fragment targeted to endoplasmic reticulum membrane and N-terminal CALR (lower panel, ER construct from (*10*)) localization in HEK293 cells. Red: Actin, green: Streptavidin and blue: DAPI, nuclear staining. StA: Streptavidin. Right: Membrane construct positively correlates with the actin signal, while the ER does not. Data were analyzed by unpaired Student's t-test. **P=0.0018. (C) Positive correlation between the CDH2-CALR SPLIT-Turbo ID and immunolabeled Calreticulin. Data were analyzed using the *Coloc2* Plugin software in FijiJ (D) Calr-inducers promote the Calr expression in vivo (zebrafish embryos). (E) Cumulative interaction fraction observed in $ROS^{dependent}$ and $ROS^{independent}$ compounds. Data were analyzed using one-way ANOVA. $P<0.0001$. (F) Calr-inducers do not promote apoptosis (in zebrafish embryos). In orange we highlight the exception. (G) *calr3b* crispant showed lower *calr3b* expression.(H) Macrophage-HSPC interaction induced by the chemicals depends on *calr3b*. Legend in blue highlights the $ROS^{independent}$ compounds. (I) Surface CALR on human CD34+ cells upon $ROS^{dependent}$ compounds treatment with (Light bars) or without DPI (darker bars), an antioxidant. Data were analyzed by Kruskal-Wallis test followed by Dunn's multiple comparison. *$P=0.05$. Data are means ± SEM. (J) JC-1 staining after treatment with $ROS^{dependent}$ compounds showed impaired mitochondrial membrane potential. (K) Representative images of human CD34 cells treated with the dooming compounds revealed impaired mitochondrial membrane potential and morphology. (L) Quantification of (K). (M) Representative image showing the accumulation of mitochondrial ROS. (N) Quantification of mt-ROS upon macrophage depletion (*irf8* -/-) and *calr3a* knockout in 3 dpf zebrafish embryos. Data were analyzed by Kruskal-Wallis test followed by Dunn's multiple comparison. ****$P<0.0001$. Data are means ± SEM.

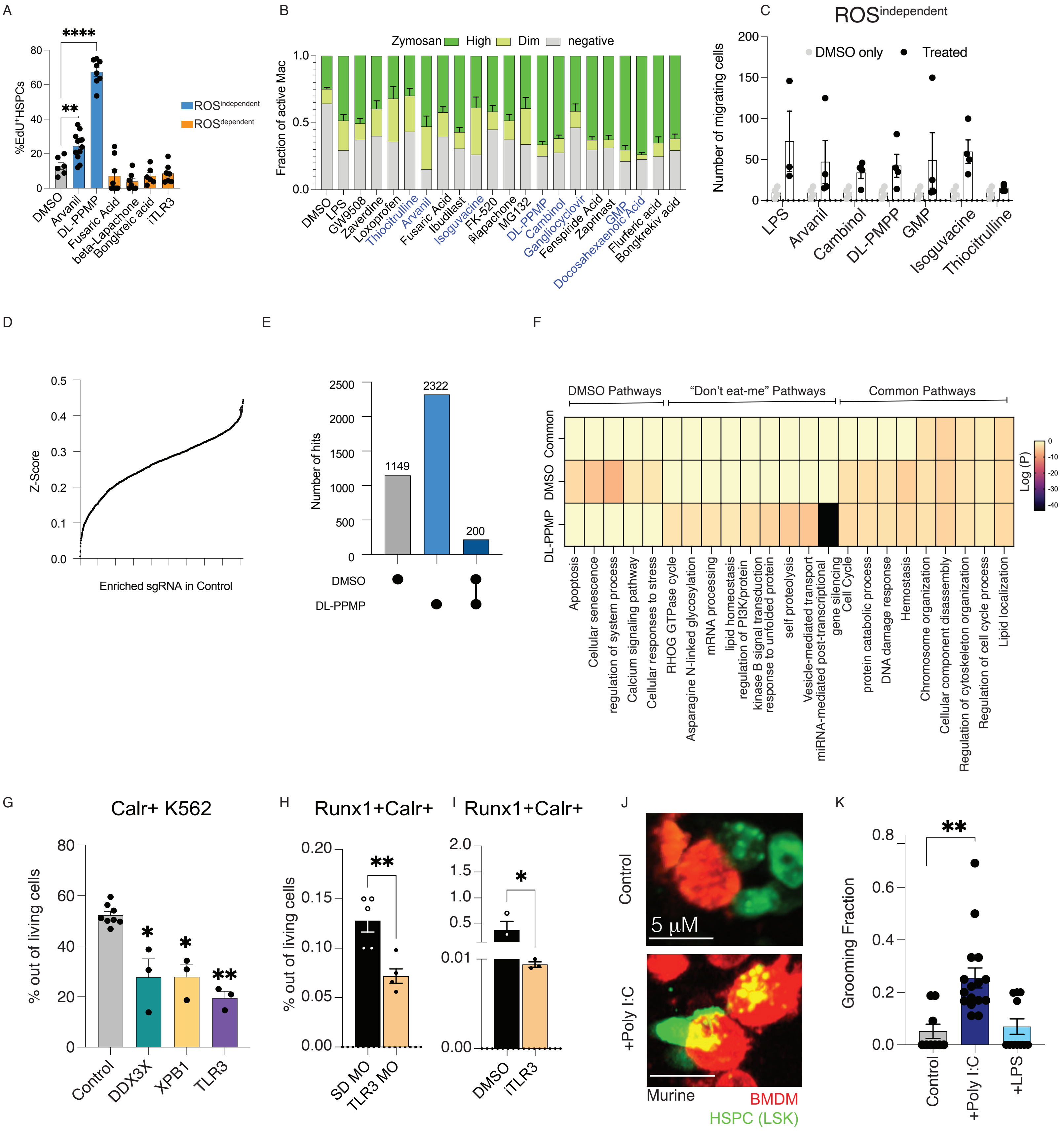

Supplementary figure 2

Fig. S2. CRISPR/Cas9 knock out screen identified TLR3 as a surface CALR mediator in the "don't eat-me" context. (A) EdU staining of *runx1+23:mCherry* embryos treated with either ROS^{independent} or ROS^{dependent} Calr-inducers identifies a significant increase in proliferation HSPCs at 3 dpf in the ROS^{independent} group. Blue bars depict the ROS^{independent} and orange bars represent the ROSdependent and iTLR3. Data were analyzed by Kruskal-Wallis test followed by Dunn's multiple comparison. $^{**}P$=0.0093, $^{***}P<0.0001$. (B) Live-cell imaging of the macrophage, Raw274 cell line, showing the macrophage phagocytosis capacity against zymosan. The green gradient depicts the intracellular zymosan intensity. (C) RAW-274 image tracking after ROS^{independent} treatment confirms the normal macrophage function. (D) Cumulative plot showing the Z-Score of the enriched sgRNA in the absence of stimulation. (E) Up-set venn diagram showing the number of significant (P<0.05) sgRNA hits. (F) Pathway enrichment analysis for sgRNAs significantly enriched in each group. (G) CRISPR/Cas-9 knockout of *DDX3X, XPB1* and *TLR3* confirmed the targets identified in the screen. Data were analyzed by Kruskal-Wallis test followed by Dunn's multiple comparison. $^{*}P$=0.04 (*DDX3X*), $^{*}P$=0.03 (*XPB1*), $^{**}P$=0.0021. Screen and validation were performed in K652 cells. (H) Morpholino *TLR3*-depleted embryos showed fewer surface Calreticulin HSPCs. Data were analyzed by unpaired Mann Whitney test. $^{*}P$=0.03. (I) 2 dpf embryos were treated with the TLR3 inhibitor (CUCPT4a, iTLR3) showed fewer surface Calreticulin HSPCs. Data were analyzed by unpaired Student's t-test. $^{**}P$=0.0034. Shapiro-Wilk test was used in (J) and (K) to analyze the data normality. (L) Representative image of the live cell imaging of murine LSK+ (green) co-cultured with autologous murine bone marrow derived macrophage (BMDM). (M) Pretreating HSPCs (LSK+) cells with Poly I:C promotes grooming behavior. Data were analyzed by Kruskal-Wallis test followed by Dunn's multiple comparison. $^{**}P$=0.0013. LPS: Lipopolysaccharides (TLR4 agonist). "+" indicates treatment. Data are means ± SEM.

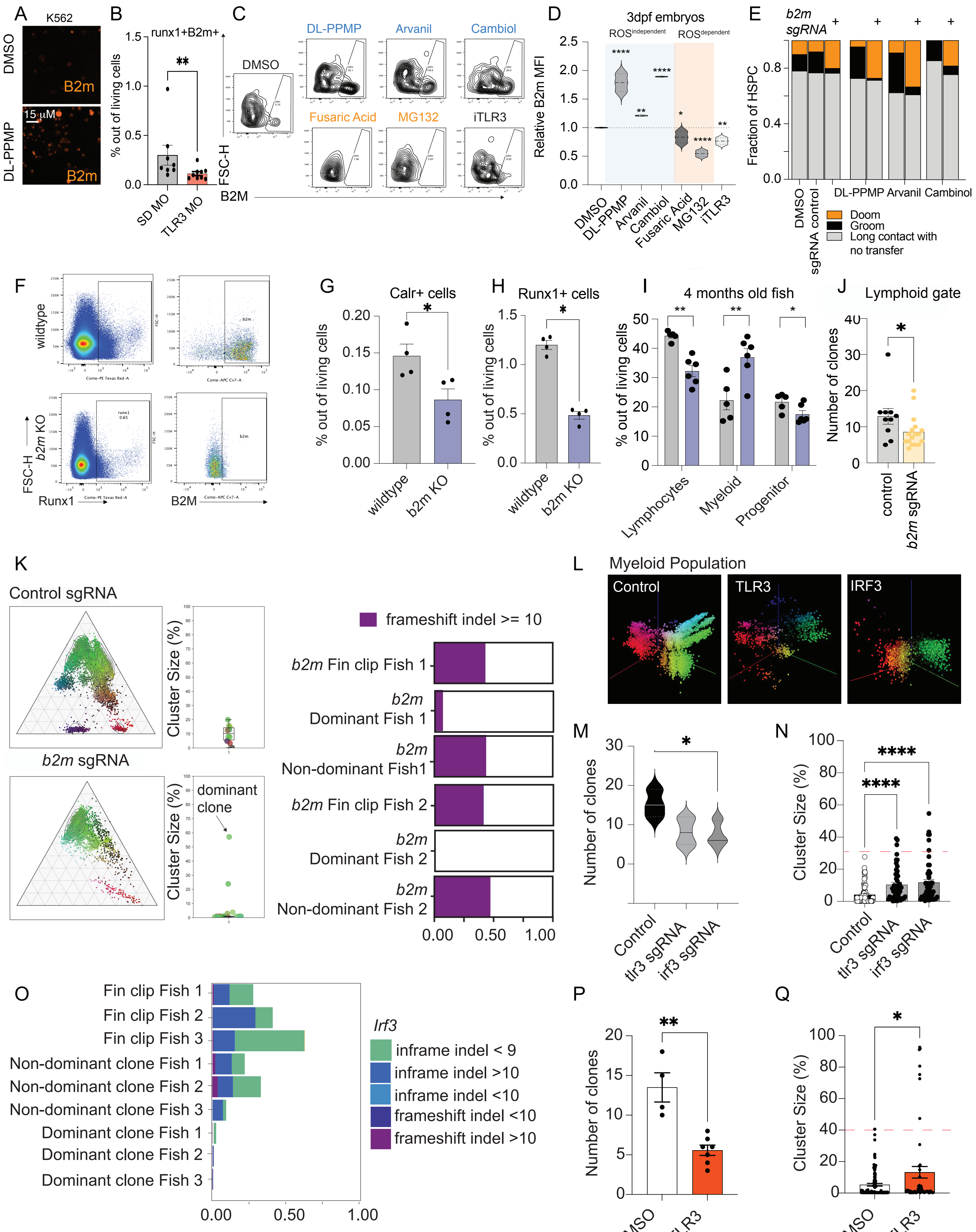

Supplementary figure 3

Science
AAAS

Fig. S3. B2m regulates HSC clonality via the dsRNA-Tlr3, Irf3 network. (A) Representative immunofluorescence image of B2M (orange) from DMSO or DL-PPMP treated K562 cells. (B) FACS analysis identifies a significant decrease in HSPC (*runx1+23:mCherry*) decreased upon TLR3 morpholino depleted embryos. Data were analyzed by Mann Whitney test. ***P*=0.0017. (C-D) $ROS^{independent}$ compounds promoted the surface B2M expression, whereas $ROS^{dependent}$ and iTLR3 failed to do the same. (E) $ROS^{independent}$ facilitated the dooming behavior upon *b2m* knock down. Data were quantified having the total number of interactions as the denominator. (F) B2m levels by FACS of wildtype and B2m stable knockout zebrafish line (*b2m* KO). (G) *b2m* KO cells from 3 dpf zebrafish embryos show lower surface Calreticulin. (H) FACS analysis shows that *b2m* KO has fewer HSPCs (runx1+ cells). (I) FACS quantification of the kidney marrow cells in wildtype (gray) and *b2m* KO (purple) from 4 months old zebrafish. (J) TWISTR-*b2m* zebrafish show fewer lymphoid clones. Data were analyzed by Mann-Whitney test. **P*=0.03 (K) Ternary diagram and cluster clone size percentage from TWISTR-control and TWISTR-*b2m* adult zebrafish (left panel). CRISPRVar analysis identifies that the dominant clones are composed by wildtype cells that resisted the macrophage dooming, while non-dominant clones harbors *b2m* CRISPR-editing. (L) 3D Zbow analysis. (M) Number of clones found by zbow analysis. Data were analyzed byKruskal-Wallis test followed by Dunn's multiple comparison test. **P*=0.03. (N) Cluster size percentage measured by the zbow analysis. Data were analyzed byKruskal-Wallis test followed by Dunn's multiple comparison test.*****P<0.0001*. (O) CRISPRVar analysis identifies that the dominant clones are composed by wildtype cells that resisted the macrophage dooming, while non-dominant clones harbors *irf3* CRISPR-editing. (P) Number of clones found by zbow analysis after treating 2 dpf embryos with iTLR3. Data were analyzed by Mann-Whitney test. ***P*=0.0012. (Q) Cluster size percentage measured by the zbow analysis after treating 2 dpf embryos with iTLR3. Data were analyzed by Mann-Whitney test. ***P*=0.003. Data are means ± SEM.

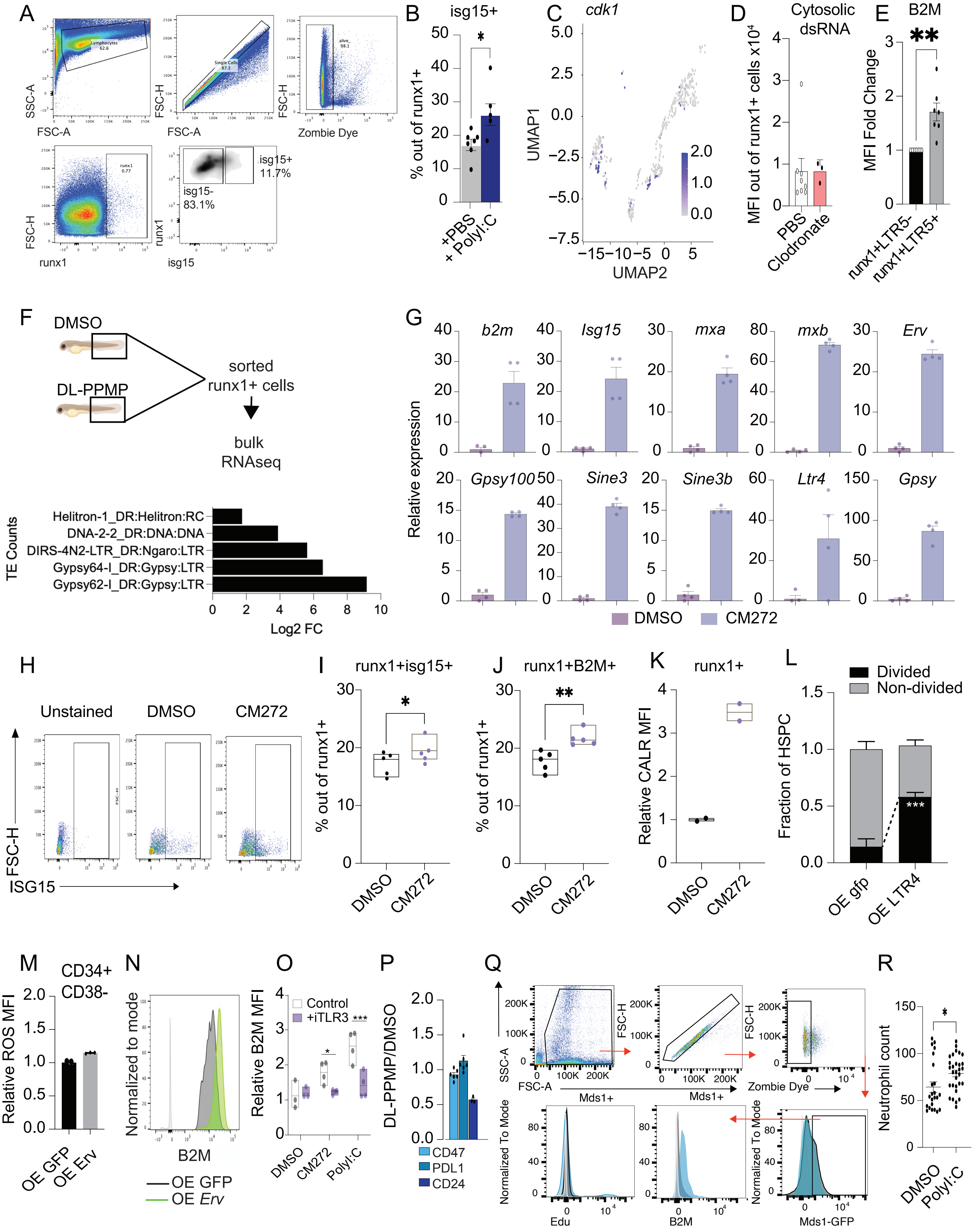

Supplementary figure 4

Fig. S4. Endogenous retrovirus is the TLR3 ligand that regulates B2m expression. (A) Representative imaging showing the isg15 levels in runx1+ cells in zebrafish embryos. (B) FACS analysis shows that embryos treated with Poly I:C have higher frequency of isg15+ HSPCs. Data were analyzed by unpaired Student t test. *P=0.03. (C) Ctlr MO enriched clusters are marked by *cdk1*. The spectral scale reports z-score. Original dataset from Watrus et. al. 2021, where *runx1*$^{+}$ were sorted into 384 plates followed by SORT-seq library preparation. (D) Flow cytometry shows equal cytosolic content of dsRNA (J2 antibody) in wildtype and macrophage-depleted embryos. (E) Significantly upregulated TE in runx1+ treated with DL-PPMP. (F) Flow cytometry shows LTR5+ HSPCs have higher B2m levels. Data were analyzed by unpaired Student t test. ***P=0.0007. (G) RT-qPCR for *b2m*, repetitive elements, *isg15, mxa* and *mxb* in CM272 3 dpf zebrafish embryos. Expression was normalized to *Rpbl3*. (H) Flow cytometry shows increased isg15+ HSPCs after CM272 treated 3 dpf zebrafish embryos. (I) Flow cytometry shows that CM272 treatment leads to higher isg15+HSPCs, (J) B2m+HSPCs and (K) surface Calr. (L) Fraction of HSPCs that divided after interacting with a macrophage. Showing that embryos overexpressing *Ltr4* under the runx1+23 enhancer have increased division ratio compared to GFP overexpressing embryos under the *runx1+23* enhancer. Data were analyzed by Kruskall-Wallis test followed by Dunn's; *P***<0.001.* Infusion technology was used to clone the *Ltr4*. OE: Overexpression. (M) Flow cytometry shows that endogenous retrovirus does not influence the ROS levels. (N) Representative histogram showing B2M levels in GFP and *Erv* overexpress human CD34+. (O) Human CD34+ cells treated with CM272 or Poly I:C lead to higher B2M levels, which is abrogated in the presence of a TLR3/dsRNA inhibitor. (P) Fold change of other "don't eat-me" molecules presented upon DL-PPMP treatment. (Q) Representative gating strategy for the levels of B2m and Edu in LT-HSC (Mds1+ cells) (R) Mpx+ (neutrophils) quantification upon Poly I:C treatment. Data were analyzed by unpaired Student t test. *P=0.05. Data are means ± SEM.

A

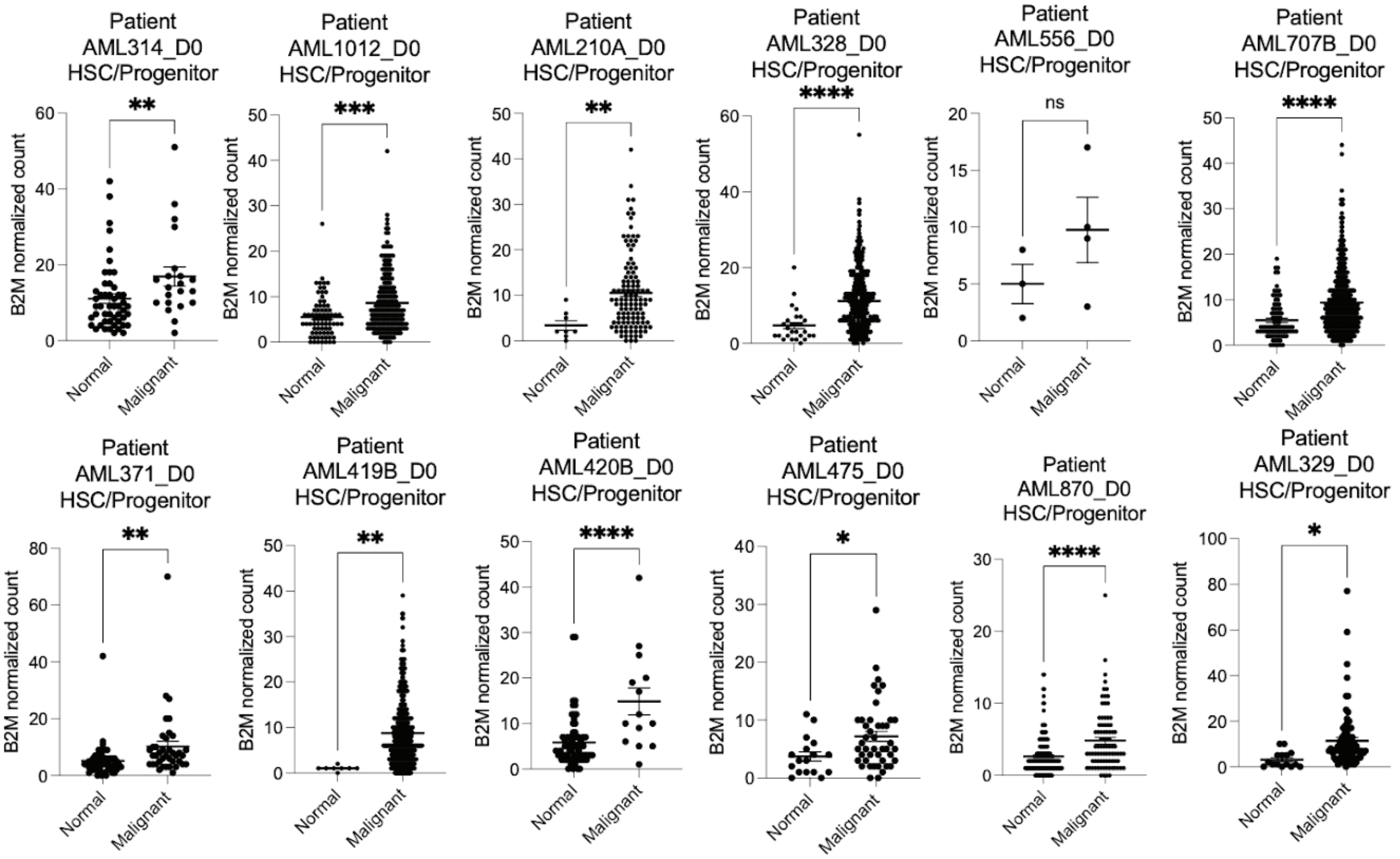

B

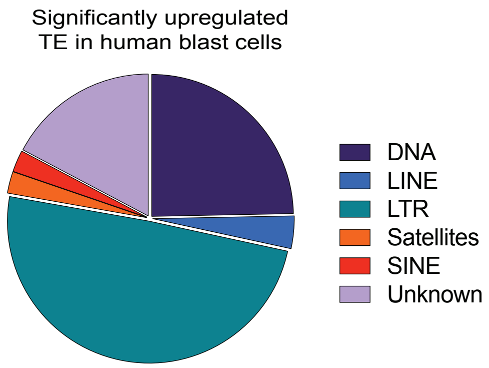

Supplementary figure 5

Fig. S5. Malignant HSPCs from AML patients showed higher *B2m* and TE expression. (A) scRNAseq quantification of *B2m* expression in HSC/Progenitor cells from normal versus malignant characterization. Each scatter plot represents a patient. Original dataset from (*62*). Data were analyzed by unpaired Student t test. (B) Pie-chart showing the significant upregulated TE found in AML-Blast cells compared to healthy HSCs. Original dataset from (*63*).

Movie S1.
ROS+runx1+ cell interacting with macrophages. Green: Mpeg; Red: Runx1; White: CellRox.

Movie S2.
ROS^{independent} compound, DL-PPMP, facilitating grooming. Red: Mpeg; Green: Runx1.

Movie S3.
Standard MO CHT. Blue: Mpeg; Red: Runx1.

Movie S4
Tlr3 MO CHT. Blue: Mpeg; Red: Runx1.

Movie S5
iTlr3 treated CHT. Blue: Mpeg; Red: Runx1.

Movie S6
Isg15+HSPC interacting with a macrophage. Blue: Mpeg; Red: Runx1; Green: Isg15.

Movie S7
DMSO treated embryos. Blue: Mpeg; Red: Runx1; Green: Isg15.

Movie S8
CM272 treated embryos. Blue: Mpeg; Red: Runx1; Green: Isg15.

Data File S1.

Excel document containing the name of the 93 Calr inducers (Tab1), the compounds that also promote higher interaction ratios (Tab2) and the statistical analysis for the macrophage-HSPCs interaction evaluation.

Data File S2.

Excel document containing the MAGECK result for the CRISPR-Cas9 screen, guides found in the DMSO group (Tab1), DL-PPMP treated cells (Tab2) and CALR/TLR3 enrichment values for DL-PPMP and Bongkreic acid (Tab3).

Data File S3.

Excel document containing the reagents, primers, sgRNAs and oligos used in this study.
